# Close congruence between Barcode Index Numbers (bins) and species boundaries in the Erebidae (Lepidoptera: Noctuoidea) of the Iberian Peninsula

**DOI:** 10.3897/BDJ.5.e19840

**Published:** 2017-08-08

**Authors:** Antonio S. Ortiz, Rosa M. Rubio, Juan J. Guerrero, Manuel J. Garre, Jose Serrano, Paul D.N. Hebert, Axel Hausmann

**Affiliations:** 1 Universidad de Murcia, Murcia, Spain; 2 Biodiversity Institute of Ontario, Guelph, Canada; 3 Bavarian State Collection of Zoology, München, Germany

**Keywords:** barcode library, CO1, Lepidoptera, DNA barcoding, Spain, Iberian Peninsula, mitochondrial DNA

## Abstract

The DNA barcode reference library for Lepidoptera holds much promise as a tool for taxonomic research and for providing the reliable identifications needed for conservation assessment programs. We gathered sequences for the barcode region of the mitochondrial cytochrome c oxidase subunit I gene from 160 of the 176 nominal species of Erebidae moths (Insecta: Lepidoptera) known from the Iberian Peninsula. These results arise from a research project which constructing a DNA barcode library for the insect species of Spain. New records for 271 specimens (122 species) are coupled with preexisting data for 38 species from the Iberian fauna. Mean interspecific distance was 12.1%, while the mean nearest neighbour divergence was 6.4%. All 160 species possessed diagnostic barcode sequences, but one pair of congeneric taxa (*Eublemma rosea* and *Eublemma rietzi*) were assigned to the same BIN. As well, intraspecific sequence divergences higher than 1.5% were detected in four species which likely represent species complexes. This study reinforces the effectiveness of DNA barcoding as a tool for monitoring biodiversity in particular geographical areas and the strong correspondence between sequence clusters delineated by BINs and species recognized through detailed taxonomic analysis.

## Introduction

The Mediterranean peninsulas of Iberia, Italy, and the Balkans are important hotspots of biodiversity (Myers et al. 2000) as they support more genetic and species diversity than higher latitudes in Europe. The Mediterranean biota, however, has been impoverished by human impacts over a long period of time, which have completely transformed the region (Mooney 1988), so that their habitats are now greatly challenged. A variety of taxa including freshwater fishes, amphibians, and lizards show genetic and phylogeographic concordances indicating that the Iberian Peninsula was a refugial region with many endemic species, as would be expected from long-term refugia fostering speciation through divergence of separate lineages (Gómez and Lunt 2007; Hewitt 2011). In fact, Oosterbroek (1994) suggested that the Mediterranean region, together with the Caucasian region and the Far East, are the most species-rich areas of the Palearctic. The insect fauna of the Mediterranean includes about 75% of the Western Palearctic fauna (Balleto and Casale 1991).

Lepidoptera is one of the most species-rich orders of insects, with some 155,000 described species found in diverse habitats from cooler regions to tropical forests (Pogue 2009, Nieukerken et al. 2011). In the Palearctic region, almost 25,000 species have been described, including some 8,000 species of Macroheterocera belonging to the superfamilies Geometroidea, Drepanoidea, Bombycoidea, Sphingoidea and Noctuoidea (Konstantinov et al. 2009). Among the 1,577 species of macroheterocerans known from the Iberian Peninsula, almost 20% of Palearctic fauna, 881 species belong to Noctuoidea (Vives 2014) with approximately 5% of these taxa endemic to this region. Five families (Notodontidae, Erebidae, Nolidae, Euteliidae and Noctuidae) of Noctuoidea are represented with the Erebidae including 176 species (21%) in the subfamilies Arctiinae (64), Erebinae (40), Eublemminae (21), Lymantriinae (17), Herminiinae (14) and some others (20).

Since DNA barcodes were proposed as a tool for species identification (Hebert et al. 2003), early studies indicated that DNA barcode libraries require comprehensive coverage of known species to enable the identification of newly collected specimens (Ekrem et al. 2007). Many studies have now employed barcodes to monitor lepidopteran biodiversity (e.g.: Janzen et al. 2005, Janzen et al. 2009, Lukhtanov et al. 2009, Dinca et al. 2010, deWaard et al. 2011, Hausmann et al. 2011a, Hausmann et al. 2011b, Hausmann et al. 2013, Hausmann et al. 2016b, Huemer 2012, Wilson et al. 2013, Huemer et al. 2014, Liu et al. 2014, Zahiri et al. 2014, Miller et al. 2016), solving systematic problems (e.g.: Hajibabaei et al. 2006, Burns et al. 2008, Hausmann 2011, Hausmann et al. 2009a, Hausmann et al. 2009b, Hausmann et al. 2014, Hausmann et al. 2016a, Huemer and Mutanen 2012, Hundsdoerfer et al. 2009, Mutanen et al. 2012) and to detect invasive species (e.g.: Armstrong and Ball 2005, Ball and Armstrong 2006, deWaard et al. 2010, Nagoshi et al. 2011, Mastrangelo et al. 2014). Several studies have shown that 95-100% of the species in regional faunas can be discriminated with DNA barcodes (Hajibabaei et al. 2006, Hebert et al. 2009, Hausmann 2011, Hausmann et al. 2011a, Hausmann et al. 2011b, Hausmann et al. 2013). Recent work has tested the impact on barcode resolution of expanding from a regional to continental scale (Mutanen et al. 2012, Hausmann et al. 2013, Huemer et al. 2014, Dinca et al. 2015). Barcodes discriminated all 75 Australian species in the family Sphingidae regardless of their collection site (Rougerie et al. 2014), while 1000 species of Lepidoptera shared by Austria and Finland showed a small decline in identiﬁcation success when identification was based on a barcode records from just one of these locales (Huemer et al. 2014). However, the application of DNA barcoding requires the construction of a complete reference library and the subsequent assessment of its efficacy for discriminating species. Particularly interesting are taxa for which barcode results are discordant with current taxonomy as they may reflect overlooked cryptic species, species that hybridize, cases of synonymy or situations where a secondary barcode marker is required for species diagnosis.

The Iberian macromoth fauna has been well studied taxonomically and ecologically, reflecting its occupation of a peninsular refuge and a bridge between Europe and Northern Africa. This project represents the first in a series that will assemble a DNA barcode library for all macromoth species from the Iberian Peninsula because its species richness and genetic diversity are the highest in Europe.

The present study has the primary goal of providing access to a comprehensive barcode library for the Erebidae species of the Iberian Peninsula. We additionally test how the molecular delineation of COI (mitochondrial cytochrome *c* oxidase subunit I) barcode haplotype clusters compares with the morphological species concept are useful tools for assessing biodiversity and indicating the completeness of biotic surveys. Such data releases in the Barcode of Life Datasystem (BOLD) and GenBank help to democratize access to biodiversity information because each barcode record is accompanied by georeferenced data and images of its source specimen (Ratnasingham and Hebert 2007, Janzen et al. 2009, Hebert et al. 2009, Ratnasingham 2016). Ratnasingham and Hebert (2013) recently implemented the Barcode Index Number (BIN) system as a registry for all species records on BOLD. BINs are important when automated recognition performs well for groups whose taxonomy is as accurately known as Lepidoptera because it helps to refine current species determinations based on morphology to accurately assign unknown samples to an existing species in BOLD. However, it also provides a first estimate of species diversity in groups where the taxonomic framework is missing or poor. Although the BIN system is potentially of great importance to taxonomic research, its performance has seen limited examination.

The specific aims of this study are (a) to present a public data release of DNA barcodes for Iberian Erebidae, (b) to critically analyse intraspecific variation and interspecific distances in the barcode region and how they relate to traditionally recognized species and, (c) to test the correspondence between BINs and traditionally recognized species.

## Material and methods

### Sampling

Specimens were sampled across Spain and the Canary Islands. Permission to collect Lepidoptera in Spain is required both inside and outside nature reserves in all regions. This study considers all 13 subfamilies of Erebidae known from Iberian Peninsula, representing 176 species (Vives 2014 with some modifications and fauna-eu.org accessed at July, 2017). All specimens were identified by the authors, and identifications were confirmed by dissection in all difficult cases. Iberian specimens are deposited in the Research Collection of Animal Biology (RCBA) at the Department of Zoology and Physical Anthropology of the Universidad of Murcia (Spain). Taxonomy and nomenclature of families, genera and species follow Fauna Europaea (Fibiger and Skule 2013). For further details on specimens see Suppl. material 1.

DNA barcodes were obtained by sampling a dry leg from each of a few vouchers per species, trying to include material from all Iberian faunal regions. In total, tissue samples from 271 Iberian specimens (including one Canarian), representing 122 of the species present in the Iberian Peninsula were submitted for analysis. In addition, existing sequence records were included for 38 of the 54 missing species, adding 87 sequences. These samples derived from Germany (67 sequences; 25 species), Italy (13 seqs; 9 spp.) France (2 seqs; 1 sp.), Cyprus, Ethiopia, Hungary, Macedonia and Russia (each 1 seq.; 1 sp.).

### DNA Analysis

PCR amplification and DNA sequencing were performed at the Canadian Centre for DNA Barcoding following standard high-throughput protocols (Ivanova et al. 2006, deWaard et al. 2008), that can be accessed under www.dnabarcoding.ca/pa/ge/research/protocols. PCR amplification with a single pair of primers consistently recovered a 658 bp region near the 5’ terminus of the mitochondrial cytochrome c oxidase I (COI) gene that included the standard 648 bp barcode region for the animal kingdom (Hebert et al. 2003). All barcoded voucher specimens are listed in Suppl. material 1 and Suppl. material 2. DNA extracts are currently stored at the Canadian Centre for DNA Barcoding. All new sequences were deposited in GenBank according to the data release policy of the International Barcode of Life Project, and accession numbers are given in Suppl. material 1. Complete specimen data including images, voucher deposition, GenBank accession numbers, GPS coordinates, sequence and trace files can easily be accessed in the Barcode of Life Data System (Ratnasingham and Hebert 2007, Ratnasingham 2016) in the dataset DS-IBEREBID (https://doi.org/10.5883/DS-IBEREBID). Access has been restricted until 2018 for a very few species that show deep intraspecific divergences to enable additional studies aimed at clarifying their taxonomic status.

### Data Analysis

Sequence divergences for the barcode region were quantified using the Kimura 2 Parameter distance model, employing the analytical tools in BOLD (BOLD alignment, pairwise deletion). Genetic distances between species are reported as minimum pairwise distances, while intraspecific variation is reported as mean and maximum pairwise distances.

Each specimen with a sequence longer than 500bp (listed in Suppl. material 1, similarities visualized in a Neighbor Joining tree, Suppl. material 4, records analysed in May 2016) automatically gained a BIN assignment on BOLD. BINs are generated using the Reﬁned Single Linkage (RESL) algorithm which employs a three-phased analysis to reach decisions on the number of BINs (= OTUs) in the overall sequence data set on BOLD (Ratnasingham and Hebert 2013). In contrast to some other approaches employed for OTU designation, such as Automatic Barcode Gap Discovery (Puillandre et al. 2012), its outcome is deterministic. It is also much faster than other approaches, such as the generalized mixed Yule-coalescent model (Pons et al. 2006, Fujisawa and Barraclough 2013), a critical requirement for the analysis of large data sets (see Ratnasingham and Hebert 2013 for of algorithm details and comparisons). Because BIN assignments are dynamically updated as new records are added to BOLD, BINs may be merged when genetically intermediate specimens are encountered or split when new records reveal clear structure in the patterns of sequence divergence. A nomenclature system, based on a set of simple rules (Ratnasingham and Hebert 2013), has been implemented in BOLD to make changes in assignments straightforward to trace and easy to understand. Whenever a discrepancy was found between DNA barcode results and a species asignement, the specimen was re-examined to confirm that its identification was correct, and that sequence results were secure.

## Results

### Traditional and BIN species delineations

Sequences were recovered friom 271 of the 304 (89.5%) specimens. All sequences were longer than 500 bp, meeting the length requirement for DNA barcode status (Ratnasingham and Hebert 2007). These results provided coverage for 124 species that were assigned to 127 BINs. Data for 36 more Iberian species were based on specimens from other regions of Europe and *Tathorhynchus exsiccata* from Ethiopia (see Suppl. material 1). Thus, 360 barcode records were available for 160 species, 90.9% of the Iberian fauna. As a 307 bp sequence was available for *Odice suava*, only 16 species lack coverage (see Suppl. material 3).

The 160 morphological species were assigned to 163 BINs and could be separated into three categories. Most (96.3%) taxa showed a perfect match between morphological species and BINs (154 species). Four (2.5%) species were each placed into two BINs, while just two species, *Eublemma rosea* and its allopatric congener *Eublemma rietzi*, were merged into the same BIN. However, even in this case, shallow barcode divergence (2.02%) allows the discrimination of *E. rietzi*, described in 2010, from the similar but morphologically separable *E. rosea.*

**Figure 1.**
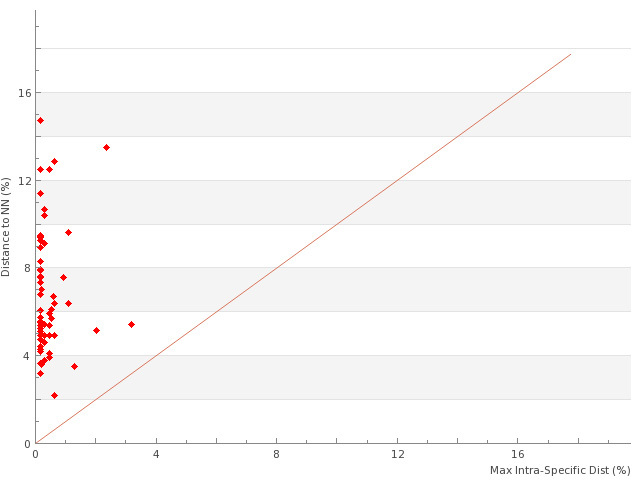
The barcode gap for 121 species of Iberian Erebidae with two or more individuals sampled is shown by plotting maximum intraspeciﬁc divergence against nearest-neighbour distance. Points above the diagonal indicate species with a barcode gap.

Considering all specimens from all species in the 13 subfamilies, Iberian Erebidae showed a mean interspecific genetic distance of 12.1% (SE <0.01; n=61,901 comparisons of barcodes >500bp). By comparison, congeneric species averaged 6.7% divergence (SE <0.01; n=1,831), while the mean nearest neighbour divergence was 6.4% (n=160). Mean and maximum intraspecific variation were 0.6% and 4.1% respectively, based upon traditionally delimited species including the four assigned to more than one BIN (n=122 species represented by more than one specimen). By comparison, the mean and maximum intra-BIN variation were 0.21% and 3.2% respectively (n=165 BINs represented by more than one specimen; SE=0.01). As a consequence, there was a clear barcode gap for almost all the species (Fig. 1).

### Species assigned to multiple BINs

Although most Iberian Erebidae showed very limited intraspecific barcode variation, 4 of the 160 species (see Suppl. material 1) were placed in two BINs, typically with more than 1.5% sequence divergence. Maximum sequence divergences between the BINs assigned to a currently recognized species averaged 2.22%, but ranged from 1.61% to 3.09%. Of the 8 BINs represented, six (75%) involved a single Iberian specimen distant from the cluster formed by its conspecifics (cf. Suppl. material 1). Interestingly, two of these six singletons involved a haplotype also detected in specimens collected outside the Iberian Peninsula.

Among the four species assigned to more than one BIN, 4 of the 8 intraspecific BIN clusters represent cases where both BINs occur in sympatry as defined by cases where the minimum geographic distance between members of the two BINs was less than 100 km. Two cases of BIN splits corresponded to subspecies recognized by traditional taxonomy: *Ocnogyna z. zoraida* (BOLD:ACE3052) & *Ocnogyna z. hemigena* (Graslin, 1850) (BOLD:AAY5665), and *Arctia v. villica* (BOLD:ACP7477) & *A. v. angelica* (Boisduval, 1829) (BOLD:ABY6789). However, no morphological differences (e.g. wing colour, wing pattern, morphology of genitalia) were evident between members of different BINs in the other two species (*Orgyia dubia, Ocnogyna baetica*). However, both cases involved geographically isolated lineages with the minimum geographic distance between members of the two BINs being around 125 km in *O. dubia* and 330 km in *O. baetica*.

## Discussion

### Identification accuracy

Following the definition of ‘diagnostic’ barcode clusters including those with monophyletic intraspecific splits (Hausmann et al. 2013), DNA barcodes discriminate all of the 160 Erebidae species examined in this study. These results are similar to those reported in other regions; 99% of the Lepidoptera from north-eastern North America were found to possess diagnostic barcodes (Hebert et al. 2009), 99% of the butterflies and larger moths of Germany (Hausmann et al. 2011b), 98.8% of the Lepidoptera species shared by two localities in Finland and Austria (Huemer et al. 2014), 98.5% of Bavarian geometrids (Hausmann et al. 2011a), 93.1% of Iberian butterflies (Dinca et al. 2015), 93% of European geometrids (Hausmann et al. 2013) and 90% of Romanian butterflies (Dinca et al. 2010).

The success of re-identification by DNA barcoding remains high in Erebidae even when analysis is extended to a European scale. A different pattern with lower number of BIN-species-matches species was reported for European Geometridae (Hausmann et al. 2013) and on a group of European leaf-mining moths (Nieukerken et al. 2012). Interestingly, geographical barcode differentiation played a minor role over distances of up to 2800 km in north-eastern American Lepidoptera (Hebert et al. 2009) and up to 1600 km in European Lepidoptera (Huemer et al. 2014) or at general scale in North-American Noctuoidea (Zahiri et al. 2014), and between the Lepidoptera of European Alps, Fennoscandia and North America (Mutanen et al. 2012). Although larger sample sizes in Europe may reveal new splits and cases of barcode sharing overlooked in the present study, identification success is unlikely to show a significant decline. In particular, increased sample sizes may raise the incidence of splits, but their detection will not lower the identification success.

### Different species assigned to the same BIN

Just one pair of species shared a BIN, the recently separated *Eublemma rosea* and *E. rietzi* (Witt and Ronkay 2011). Species pairs placed in a single BIN often show shallow divergences, meaning that DNA barcodes are diagnostic and this was true for these *Eublemma*. The endemic *E. rietzi* was described in 2010 from Granada and is only known from a small area between Baza-Cúllar-to north of the Benamaurel village although the authors considered it as Atlanto-Mediterranean corotype. On the other hand, the distribution of *E. rosea* in Europe is surprisingly disjunct between a North-western population in Iberia and the rest in the Alps, southern Italian Peninsula, Slovenia, etc. This case of BIN sharing among allopatric species with slightly divergent genetic clusters suggests that these taxa represent recently separated lineages that are still undergoing genetic differentiation and incipient speciation. The evolution of morphological traits such as genitalia is generally thought to be rapid (Eberhard 1985, Shapiro and Porter 1989, Arnqvist 1998), perhaps faster than COI diversification.

A second potential case of BIN sharing involved the *Eilema / * complex as its members were allocated to one or two BINs depending on the taxonomic status of certain Iberian populations. Witt and Ronkay (2011) view *E. complana* as one of the commonest European Lithosiini, a species showing high variation in its morphology and genital features. One of its lineages, *E. c. iberica* Mentzer, 1980 was originally described as a subspecies of *E. pseudocomplana* Daniel, 1939 but was later (Ylla et al. 2010) proposed as a subspecies of *E. complana* based on certain similarities in genital morphology. As well, the presence of a pale ochreous androconial patch on the underside of the forewing suggests a closer relationship between ** and *.* However, this reduction of the androconial scales may also reflect introgression from the allopatric *E. pseudocomplana.* Our results placed all Iberian specimens of *E. complana iberica* in a single BIN (BOLD:AAB6846) that included some specimens morphologically identified as *E. pseudocomplana*, except two specimens from the Pyrenees sharing another BIN (BOLD:ABW5869) with *E. pseudocomplana* from southern Germany. This pattern suggests that *E. complana/* may represent a case of parapatry with a hybrid zone rather than true sympatry, at least in Iberian Peninsula. BIN-sharing must be considered as rare and further studies are required to detect F1 hybrids, introgression events in Iberian populations, or misidentifications revealing problems in current, morphology-based concepts about species identification. These cases of discrepancy reflect instances where the current taxonomic system is likely flawed and revealing species deserving more intensive study.

### One species assigned to two imore BINs

Because about one fifth (34/160) of the species examined in this study were represented by a single specimen, additional samples, particularly from new geographic areas and habitats will likely reveal new BINs for some Iberian Erebidae. However, the current analysis revealed BIN splits in four species (*Rivula sericealis, Eilema sororcula, Coscinia cribraria, Lygephila craccae*) represented by singletons show a different haplotype from the rest of the European populations, potentially pointing to taxonomic implications. We detected one species, *Ocnogyna baetica*, whose Iberian members were assigned to two BINs with divergence greater than 3%, in the same way that Iberian *Eublemma parva* and *Coscinia cribraria* singletons present with other conspecific Iberian populations, suggesting these require further study.

Other cases of multiple BINs involved taxa whose discrimination is sometimes uncertain. *Ocnogyna z. zoraida* and *O. z. hemigena* as well as *Arctia v. villica* and *A. v. angelica* each involve a pair of subspecies of uncertain status. Prior studies have detected specimens with intermediate characters in putative hybrid zones suggesting recent speciation with incomplete lineage sorting and introgression. These lineages occur in sympatry, and usually possess consistent differences in external appearance as noted in the literature or that were apparent from our investigation. These correlations may justify the upgrading of these taxa to a species rank though this decision requires further integrated taxonomic study.

In one BIN-split, *Orgyia dubia*, the two BINs occurred in allopatry. The presence of two barcode lineages in *O. dubia* (‘ Rambur, 1842’) with 2.25% divergence between central and southern populations might reflect divergence that arose when populations were isolated in different glacial refugia during the Pleistocene. Moreover, the Iberian needs to be compared to the nominotypical *O. d. dubia* Tauscher, 1806 from its type locality in Russia.

The genus *Setina* is considered taxonomically difficult due to high inter-populational variability in morphology. In our study, *Setina flavicans* and *S. cantabrica* showed clear divergence (2.34%) although the nearest neighbour for both taxa is *S. irrorella* (Linnaeus, 1758) with 1.77% distance. Current taxonomy views both *S. cantabrica* and *S. flavicans* (Freina and Witt 1987, Ylla et al. 2010) as distinct species, although Leraut (2006) treated the latter as a synonym of *S. irrorella*, An integrated taxonomic study is needed to clarify relationships within this genus at a European level.

Iberian *Chelis* species show how DNA barcodes divergences are often correlated with differences in morphology which can easily be overlooked or disputed (Ortiz et al. 2016). In this case, specimens were assigned to three BINs, two endemics (*C. arragonensis*, *C. cantabrica*), and a third matching one of the haplotypes of *C. maculosa*, a species which is widely distributed in Europe. Interestingly, *C. cantabrica* from the Cantabric Mountains (BOLD: ACE5195) showed closer barcode similarity (98.39%) to Alpine specimens of *C. simplonica* (Boisduval 1840) (BOLD: ABW6572) at a distance of 1,000 km than to its congeners in the Iberian Peninsula. The genetic divergences between Iberian *C. maculosa, C. arragonensis* and *C. cantabrica* reflect frequent scenarios of geographical isolation during the Quaternary (Pleistocene and late Pliocene) in the Iberian Peninsula (Ortiz et al. 2016).

The strong morphological similarity of all specimens in taxa with BIN splits further supports their recent separation although most cases may represent cryptic species complexes. In such cases, taxonomic decisions are often subjective and depend on the choice of species delimitation models, application of species concepts and taxonomic principles, especially when allopatric populations are involved (Mutanen et al. 2012). Subsequent studies need to involve more detailed morphological investigation and the inclusion of nuclear markers to assess alternative explanations for the sequence divergence patterns, such as geographic structure, biased variation induced by *Wolbachia* (Smith et al. 2012), heteroplasmy (Frey and Frey 2004) or co-ampliﬁcation of pseudogenes (Song et al. 2008).

## Conclusions

1. Sequences for the barcode region of the mitochondrial COI gene from 271 specimens (160 species) of Erebidae moths (Insecta: Lepidoptera) from the Iberian Peninsula were gathered, showing a mean interspecific distance of 12.1%, while the mean nearest neighbour divergence was 6.4%.

2. All 160 species possessed diagnostic barcode sequences, but one pair of congeneric taxa were assigned to the same BIN. Intraspecific sequence divergences higher than 1.5% were detected in four species which likely represent species complexes.

3. This study reinforces the effectiveness of DNA barcoding as a tool for monitoring biodiversity in particular geographical areas and the strong correspondence between sequence clusters delineated by BINs and species recognized through detailed taxonomic analysis.

## Supplementary Material

Supplementary material 1 Accesion numbers and BINs Data type: Genomic data
Brief description: List of species names, sample-IDs, process-IDs (from BOLD database), COI-5P bps, BINs, GenBank Accession numbers, collection country, and Institution storing vouchers. Abbreviations: BIN = Barcode Index Number. NST = Naturmuseum Suedtirol. PCEM = Private Collection of Enrique Murria Beltran. RCAH = Research Collection of Alfred Haslberger. RCBA-UMU = Research Collection Biologia Animal-Universidad de Murcia. RCBD = Research Collection of Bernard Dardenne. RCCZ = Research Collection of Christian Zehentner. RCPL = Research Collection of Peter Lichtmannecker. RCRS = Research Collection of Ralph Sturm. RCTG = Research Collection of Theo Gruenewald. TLF = Tiroler Landesmuseum Ferdinandeum. ZSM = Zoologische Staatssammlung Muenchen.
File: oo_146622.pdf Ortiz, Rubio, Guerrero, Garre

Supplementary material 2 Systematic list of subfamilies and species, barcode gap analysis (Mean and Maximum intraspecific variation and distance to nearest neighbor NN) for 160 Iberian species in the all European Erebidae Data type: Genetic diversity data
Brief description: Systematic list of subfamilies and species, barcode gap analysis (Mean and Maximum intraspecific variation and distance to nearest neighbor NN) for 160 Iberian species in the all European Erebidae
File: oo_146623.pdf Ortiz, Rubio, Guerrero, Garre

Supplementary material 3 List of 16 Iberian taxa without a BIN assignment Data type: Species list
Brief description: List of 16 Iberian taxa without a BIN assignment (awaiting DNA barcoding); a species with short sequences are marked with an *.
File: oo_146624.pdf Ortiz, Rubio, Guerrero, Garre

Supplementary material 4 BOLD TaxonID Tree Data type: Image
Brief description: Genetic similarities visualized in a Neighbor Joining tree
File: oo_146625.pdf Ortiz, Rubio, Guerrero, Garre
