# Supplementary material for: Close congruence between Barcode Index Numbers (bins) and species boundaries in the Erebidae (Lepidoptera: Noctuoidea) of the Iberian Peninsula
Source: Biodivers Data J. 2017 Aug 8;(5):e19840. doi: 10.3897/BDJ.5.e19840 (PMC5558050; doi:10.3897/BDJ.5.e19840)
Supplement: Supplementary material 1 — Accesion numbers and BINs [file bdj-05-e19840-s001.pdf]

Appendix S1: Specimens details, accession numbers and BINs.

List of species names, process-IDs (from BOLD database), sample-IDs, number of COI-5P bp, BINs, GenBank Accession numbers, country and collection voucher specimens. Abbreviations: BIN = Barcode Index Number. NST = Naturmuseum Suedtirol. PCEM = Private Collection of Enrique Murria Beltran. RCAH = Research Collection of Alfred Haslberger. RCBA-UMU = Research Collection Biologia Animal-Universidad de Murcia. RCBD = Research Collection of Bernard Dardenne. RCCZ = Research Collection of Christian Zehentner. RCPL = Research Collection of Peter Lichtmannecker. RCRS = Research Collection of Ralph Sturm. RCTG = Research Collection of Theo Gruenewald. TLF = Tiroler Landesmuseum Ferdinandeum. ZSM = Zoologische Staatssammlung Muenchen.

Identification	Process ID	Sample ID	COI-5P Seq. Length	BIN	Genbank	Country	Institution
**Arctiinae**							
Amata phegea	FBLMU073-09	BC ZSM Lep 25583	658	BOLD:AAM0522	HQ955224	Germany	ZSM
Arctia caja	IBLAO551-12	AOC Lep 00646	658	BOLD:AAA8530	KY370715	Spain	RCBA-UMU
Arctia caja	IBLAO552-12	AOC Lep 00647	658	BOLD:AAA8530	KY370713	Spain	RCBA-UMU
Arctia festiva	IBLAO554-12	AOC Lep 00649	581	BOLD:ABW9262	KY370582	Spain	RCBA-UMU
Arctia festiva	IBLAO1091-14	AOC Lep 01186	658	BOLD:ABW9262	KY370558	Spain	RCBA-UMU
Arctia villica angelica	IBLAO733-12	AOC Lep 00828	658	BOLD:ABY6789	On progress	Spain	RCBA-UMU
Arctia villica angelica	IBLAO734-12	AOC Lep 00829	658	BOLD:ABY6789	On progress	Spain	RCBA-UMU
Arctia villica angelica	IBLAO1060-14	AOC Lep 01155	658	BOLD:ACP7477	On progress	Spain	RCBA-UMU
Arctia villica angelica	IBLAO1061-14	AOC Lep 01156	658	BOLD:ACP7477	On progress	Spain	RCBA-UMU
Arctia villica angelica	IBLAO1067-14	AOC Lep 01162	658	BOLD:ABY6789	On progress	Spain	RCBA-UMU
Arctia villica angelica	IBLAO1068-14	AOC Lep 01163	658	BOLD:ABY6789	On progress	Spain	RCBA-UMU
Arctia villica angelica	IBLAO1069-14	AOC Lep 01164	658	BOLD:ABY6789	On progress	Spain	RCBA-UMU
Arctia villica angelica	IBLAO1070-14	AOC Lep 01165	658	BOLD:ABY6789	On progress	Spain	RCBA-UMU
Arctia villica angelica	IBLAO1071-14	AOC Lep 01166	658	BOLD:ABY6789	On progress	Spain	RCBA-UMU
Arctia villica angelica	IBLAO1107-14	AOC Lep 01202	658	BOLD:ABY6789	On progress	Spain	RCBA-UMU
Arctia villica angelica	IBLAO1127-14	AOC Lep 01222	658	BOLD:ABY6789	On progress	Spain	RCBA-UMU
Arctia villica angelica	IBLAO1128-14	AOC Lep 01223	658	BOLD:ABY6789	On progress	Spain	RCBA-UMU
Arctia villica villica	IBLAO553-12	AOC Lep 00648	658	BOLD:ACP7477	On progress	Spain	RCBA-UMU
Arctia villica villica	IBLAO951-14	AOC Lep 01046	658	BOLD:ACP7477	On progress	Spain	RCBA-UMU
Arctia villica villica	IBLAO952-14	AOC Lep 01047	658	BOLD:ACP7477	On progress	Spain	RCBA-UMU
Arctia villica villica	IBLAO1073-14	AOC Lep 01168	658	BOLD:ACP7477	On progress	Spain	RCBA-UMU
Arctia villica villica	IBLAO1124-14	AOC Lep 01219	658	BOLD:ACP7477	On progress	Spain	RCBA-UMU
Arctia villica villica	IBLAO1125-14	AOC Lep 01220	658	BOLD:ACP7477	On progress	Spain	RCBA-UMU
Arctia villica villica	IBLAO1126-14	AOC Lep 01221	658	BOLD:ACP7477	On progress	Spain	RCBA-UMU
Artimelia latreillii	IBLAO1100-14	AOC Lep 01195	658	BOLD:ACL5676	KY370736	Spain	RCBA-UMU
Artimelia latreillii	IBLAO1101-14	AOC Lep 01196	658	BOLD:ACL5676	KY370698	Spain	RCBA-UMU
Atlantarctia tigrina	IBLAO549-12	AOC Lep 00644	658	BOLD:AAJ1926	KY370577	Spain	RCBA-UMU
Atlantarctia tigrina	IBLAO550-12	AOC Lep 00645	658	BOLD:AAJ1926	KY370561	Spain	RCBA-UMU
Atolmis rubricollis	GWOR4226-09	BC ZSM Lep 21510	658	BOLD:AAD2377	JF415319	Germany	ZSM
Atolmis rubricollis	GWORK365-09	BC ZSM Lep 21600	658	BOLD:AAD2377	JF415317	Germany	ZSM
Atolmis rubricollis	GWORK366-09	BC ZSM Lep 21601	658	BOLD:AAD2377	JF415318	Germany	ZSM
Atolmis rubricollis	GWORA2562-09	BC ZSM Lep 31819	658	BOLD:AAD2377	HM393252	Germany	RCRS
Callimorpha dominula	GWOR3966-09	BC ZSM Lep 21250	658	BOLD:AAD5343	JF415320	Germany	ZSM

Identification	Process ID	Sample ID	COI-5P Seq. Length	BIN	Genbank	Country	Institution
Callimorpha dominula	GWORL402-09	BC ZSM Lep 22304	595	BOLD:AAD5343	GU686889	Germany	ZSM
Callimorpha dominula	GWORE2018-09	BC ZSM Lep 22416	657	BOLD:AAD5343	GU654846	Germany	ZSM
Callimorpha dominula	FBLMV287-09	BC ZSM Lep 28267	648	BOLD:AAD5343	GU707345	Germany	RCTG
Callimorpha dominula	GWORA2560-09	BC ZSM Lep 31817	658	BOLD:AAD5343	HQ957227	Germany	RCRS
Chelis arragonensis	IBLAO555-12	AOC Lep 00650	658	BOLD:AAF2636	KT381896	Spain	RCBA-UMU
Chelis arragonensis	IBLAO842-12	AOC Lep 00937	643	BOLD:AAF2636	KT381883	Spain	RCBA-UMU
Chelis arragonensis	IBLAO916-12	AOC Lep 01011	646	BOLD:AAF2636	KT381890	Spain	RCBA-UMU
Chelis cantabrica	IBLAO788-12	AOC Lep 00883	658	BOLD:ACE5195	KT381888	Spain	RCBA-UMU
Chelis cantabrica	IBLAO789-12	AOC Lep 00884	658	BOLD:ACE5195	KT381895	Spain	RCBA-UMU
Chelis cantabrica	IBLAO840-12	AOC Lep 00935	658	BOLD:ACE5195	KT381891	Spain	RCBA-UMU
Chelis cantabrica	IBLAO841-12	AOC Lep 00936	658	BOLD:ACE5195	KT381886	Spain	RCBA-UMU
Chelis maculosa	IBLAO556-12	AOC Lep 00651	658	BOLD:AAF2637	KT381882	Spain	RCBA-UMU
Chelis maculosa	IBLAO843-12	AOC Lep 00938	658	BOLD:AAF2637	KT381889	Spain	RCBA-UMU
Chelis maculosa	IBLAO919-12	AOC Lep 01014	646	BOLD:AAF2637	KT381885	Spain	RCBA-UMU
Chelis maculosa	IBLAO920-12	AOC Lep 01015	646	BOLD:AAF2637	KT381892	Spain	RCBA-UMU
Chelis maculosa	IBLAO921-12	AOC Lep 01016	646	BOLD:AAF2637	KT381887	Spain	RCBA-UMU
Chelis maculosa	IBLAO922-12	AOC Lep 01017	646	BOLD:AAF2637	KT381894	Spain	RCBA-UMU
Chelis maculosa	IBLAO923-12	AOC Lep 01018	646	BOLD:AAF2637	KT381898	Spain	RCBA-UMU
Chelis maculosa	IBLAO924-12	AOC Lep 01019	626	BOLD:AAF2637	KT381893	Spain	RCBA-UMU
Chelis maculosa	IBLAO926-12	AOC Lep 01021	646	BOLD:AAF2637	KT381881	Spain	RCBA-UMU
Chelis maculosa	IBLAO927-12	AOC Lep 01022	658	BOLD:AAF2637	KT381897	Spain	RCBA-UMU
Coscinia cribraria	IBLAO557-12	AOC Lep 00652	658	BOLD:AAD9042	KY370610	Spain	RCBA-UMU
Coscinia cribraria	IBLAO774-12	AOC Lep 00869	658	BOLD:AAD9042	KY370626	Spain	RCBA-UMU
Coscinia striata	IBLAO872-12	AOC Lep 00967	658	BOLD:AAJ5576	KY370655	Spain	RCBA-UMU
Coscinia striata	IBLAO1090-14	AOC Lep 01185	658	BOLD:AAJ5576	KY370681	Spain	RCBA-UMU
Cybosia mesomella	IBLAO855-12	AOC Lep 00950	658	BOLD:AAD1825	KY370697	Spain	RCBA-UMU
Cybosia mesomella	IBLAO871-12	AOC Lep 00966	624	BOLD:AAD1825	KY370653	Spain	RCBA-UMU
Cymbalophora pudica	IBLAO544-12	AOC Lep 00639	658	BOLD:AAG6227	KY370745	Spain	RCBA-UMU
Diacrisia sannio	IBLAO538-12	AOC Lep 00633	658	BOLD:AAB8660	KY370657	Spain	RCBA-UMU
Diacrisia sannio	IBLAO1033-14	AOC Lep 01128	658	BOLD:AAB8660	KY370699	Spain	RCBA-UMU
Diaphora mendica	IBLAO542-12	AOC Lep 00637	658	BOLD:AAB9868	KY370644	Spain	RCBA-UMU
Diaphora mendica	IBLAO790-12	AOC Lep 00885	658	BOLD:AAB9868	KY370574	Spain	RCBA-UMU
Dysauxes ancilla	FBLMU057-09	BC ZSM Lep 25567	624	BOLD:AAK9768	HM422160	Germany	ZSM
Dysauxes ancilla	FBLMU558-09	BC ZSM Lep 27208	658	BOLD:AAK9768	HQ955232	Germany	ZSM
Dysauxes punctata	LEATG255-14	TLMF Lep 14042	658	BOLD:ACM6939	KY370581	Italy	TLF
Dysauxes punctata	LEATJ207-15	TLMF Lep 18637	658	BOLD:ACM6939	KY370548	Italy	TLF
Eilema albicosta	IBLAO892-12	AOC Lep 00987	658	BOLD:ACD0672	KY370711	Spain	RCBA-UMU
Eilema caniola	IBLAO525-12	AOC Lep 00620	658	BOLD:AAF6264	KY370630	Spain	RCBA-UMU
Eilema caniola	IBLAO526-12	AOC Lep 00621	658	BOLD:AAF6264	KY370549	Spain	RCBA-UMU
Eilema complana complana	IBLAO518-12	AOC Lep 00613	658	BOLD:AAB6846	On progress	Spain	RCBA-UMU
Eilema complana complana	IBLAO519-12	AOC Lep 00614	658	BOLD:AAB6846	On progress	Spain	RCBA-UMU
Eilema complana complana	IBLAO832-12	AOC Lep 00927	658	BOLD:AAB6846	On progress	Spain	RCBA-UMU
Eilema complana complana	IBLAO928-12	AOC Lep 01023	658	BOLD:AAB6846	On progress	Spain	RCBA-UMU

Identification	Process ID	Sample ID	COI-5P Seq. Length	BIN	Genbank	Country	Institution
Eilema complana iberica	IBLAO520-12	AOC Lep 00615	658	BOLD:AAB6846	On progress	Spain	RCBA-UMU
Eilema complana iberica	IBLAO521-12	AOC Lep 00616	658	BOLD:AAB6846	On progress	Spain	RCBA-UMU
Eilema complana iberica	IBLAO823-12	AOC Lep 00918	658	BOLD:AAB6846	On progress	Spain	RCBA-UMU
Eilema complana iberica	IBLAO824-12	AOC Lep 00919	658	BOLD:AAB6846	On progress	Spain	RCBA-UMU
Eilema complana iberica	IBLAO894-12	AOC Lep 00989	658	BOLD:AAB6846	On progress	Spain	RCBA-UMU
Eilema complana iberica	IBLAO929-12	AOC Lep 01024	658	BOLD:AAB6846	On progress	Spain	RCBA-UMU
Eilema complana iberica	IBLAO930-12	AOC Lep 01025	658	BOLD:AAB6846	On progress	Spain	RCBA-UMU
Eilema complana iberica	IBLAO931-12	AOC Lep 01026	658	BOLD:AAB6846	On progress	Spain	RCBA-UMU
Eilema depressa	IBLAO917-12	AOC Lep 01012	657	BOLD:AAB6834	KY370670	Spain	RCBA-UMU
Eilema depressa	IBLAO918-12	AOC Lep 01013	658	BOLD:AAB6834	KY370679	Spain	RCBA-UMU
Eilema griseola	IBLAO533-12	AOC Lep 00628	658	BOLD:AAC1074	KY370658	Spain	RCBA-UMU
Eilema griseola	IBLAO829-12	AOC Lep 00924	658	BOLD:AAC1074	KY370728	Spain	RCBA-UMU
Eilema interpositella	IBLAO529-12	AOC Lep 00624	658	BOLD:ABW9300	KY370596	Spain	RCBA-UMU
Eilema interpositella	IBLAO530-12	AOC Lep 00625	658	BOLD:ABW9300	KY370554	Spain	RCBA-UMU
Eilema lurideola	IBLAO522-12	AOC Lep 00617	658	BOLD:AAB6833	KY370589	Spain	RCBA-UMU
Eilema lurideola	IBLAO523-12	AOC Lep 00618	658	BOLD:AAB6833	KY370595	Spain	RCBA-UMU
Eilema lutarella	IBLAO524-12	AOC Lep 00619	658	BOLD:ABZ2872	KY370684	Spain	RCBA-UMU
Eilema lutarella	IBLAO831-12	AOC Lep 00926	658	BOLD:ABZ2872	KY370700	Spain	RCBA-UMU
Eilema marcida	IBLAO1141-14	ASO Lep 1014	654	BOLD:AAK9891	KY370539	Spain	RCBA-UMU
Eilema palliatella	IBLAO513-12	AOC Lep 00608	658	BOLD:AAZ9153	KY370703	Spain	RCBA-UMU
Eilema palliatella	IBLAO514-12	AOC Lep 00609	658	BOLD:AAZ9153	KY370557	Spain	RCBA-UMU
Eilema predotae	IBLAO887-12	AOC Lep 00982	658	BOLD:ACD0670	KY370616	Spain	RCBA-UMU
Eilema pseudocomplana	IBLAO515-12	AOC Lep 00610	629	BOLD:AAB6846	On progress	Spain	RCBA-UMU
Eilema pseudocomplana	IBLAO516-12	AOC Lep 00611	658	BOLD:AAB6846	On progress	Spain	RCBA-UMU
Eilema pseudocomplana	IBLAO825-12	AOC Lep 00920	658	BOLD:ABW5869	On progress	Spain	RCBA-UMU
Eilema pseudocomplana	IBLAO826-12	AOC Lep 00921	658	BOLD:AAB6846	On progress	Spain	RCBA-UMU
Eilema pseudocomplana	IBLAO833-12	AOC Lep 00928	658	BOLD:AAB6846	On progress	Spain	RCBA-UMU
Eilema pseudocomplana	IBLAO932-12	AOC Lep 01027	658	BOLD:AAB6846	On progress	Spain	RCBA-UMU
Eilema pseudocomplana	IBLAO971-14	AOC Lep 01066	629	BOLD:ABW5869	On progress	Spain	RCBA-UMU
Eilema pseudocomplana	IBLAO1057-14	AOC Lep 01152	658	BOLD:AAB6846	On progress	Spain	RCBA-UMU
Eilema pygmaeola	IBLAO531-12	AOC Lep 00626	658	BOLD:AAE8202	KY370584	Spain	RCBA-UMU
Eilema pygmaeola	IBLAO532-12	AOC Lep 00627	658	BOLD:AAE8202	KY370672	Spain	RCBA-UMU
Eilema rungsi	IBLAO890-12	AOC Lep 00985	657	BOLD:ACD0671	KY370717	Spain	RCBA-UMU
Eilema sororcula	IBLAO830-12	AOC Lep 00925	658	BOLD:AAC1077	KY370541	Spain	RCBA-UMU
Eilema sororcula	IBLAO889-12	AOC Lep 00984	658	BOLD:AAC1077	KY370614	Spain	RCBA-UMU
Eilema uniola	IBLAO527-12	AOC Lep 00622	658	BOLD:ABW9299	KY370669	Spain	RCBA-UMU
Eilema uniola	IBLAO528-12	AOC Lep 00623	658	BOLD:ABW9299	KY370606	Spain	RCBA-UMU
Euplagia quadripunctaria	IBLAO1104-14	AOC Lep 01199	658	BOLD:AAD9583	KY370566	Spain	RCBA-UMU
Euplagia quadripunctaria	IBLAO1105-14	AOC Lep 01200	658	BOLD:AAD9583	KY370552	Spain	RCBA-UMU
Hyphoraia dejeani	IBLAO547-12	AOC Lep 00642	658	BOLD:ABW9065	KY370593	Spain	RCBA-UMU
Hyphoraia dejeani	IBLAO548-12	AOC Lep 00643	658	BOLD:ABW9065	KY370618	Spain	RCBA-UMU
Hyphoraia testudinaria	IBLAO546-12	AOC Lep 00641	658	BOLD:ABZ2799	KY370573	Spain	RCBA-UMU

Identification	Process ID	Sample ID	COI-5P Seq. Length	BIN	Genbank	Country	Institution
Lithosia quadra	IBLAO536-12	AOC Lep 00631	658	BOLD:AAB8723	KY370619	Spain	RCBA-UMU
Lithosia quadra	IBLAO537-12	AOC Lep 00632	658	BOLD:AAB8723	KY370590	Spain	RCBA-UMU
Miltochrista miniata	IBLAO511-12	AOC Lep 00606	658	BOLD:AAC4781	KY370741	Spain	RCBA-UMU
Miltochrista miniata	IBLAO512-12	AOC Lep 00607	658	BOLD:AAC4781	KY370537	Spain	RCBA-UMU
Nudaria mundana	FBLMV702-09	BC ZSM Lep 28682	658	BOLD:AAD8574	GU707282	Germany	ZSM
Ocnogyna baetica	IBLAO754-12	AOC Lep 00849	647	BOLD:ABW9505	KY370563	Spain	RCBA-UMU
Ocnogyna baetica	IBLAO1138-14	AOC Lep 01233	658	BOLD:ACL5471	KY370602	Spain	RCBA-UMU
Ocnogyna hemigena	LNOUB020-10	CLV107710	657	BOLD:AAM4054	HQ571778	Spain	PCEM
Ocnogyna zoraida	LNOUB019-10	CLV107610	657	BOLD:ACE3052	HQ571777	Spain	PCEM
Paidia rica	IBLAO972-14	AOC Lep 01067	658	BOLD:ACL5459	KY370743	Spain	RCBA-UMU
Paidia rica	IBLAO1123-14	AOC Lep 01218	658	BOLD:ACL5459	KY370685	Spain	RCBA-UMU
Parasemia plantaginis	IBLAO856-12	AOC Lep 00951	658	BOLD:AAB6883	KY370550	Spain	RCBA-UMU
Parasemia plantaginis	IBLAO857-12	AOC Lep 00952	658	BOLD:AAB6883	KY370678	Spain	RCBA-UMU
Pelosia muscerda	GWORO775-09	BC ZSM Lep 30467	658	BOLD:AAD5281	GU688499	Germany	ZSM
Pelosia muscerda	GWORO776-09	BC ZSM Lep 30468	658	BOLD:AAD5281	GU688500	Germany	ZSM
Pelosia muscerda	GWORA2564-09	BC ZSM Lep 31821	512	BOLD:AAD5281	HM393253	Germany	RCRS
Pelosia obtusa	GBLAA1109-15	BC ZSM Lep 86615	658	BOLD:AAQ2315	KY370694	Germany	ZSM
Phragmatobia fuliginosa	IBLAO543-12	AOC Lep 00638	658	BOLD:AAA6178	KY370720	Spain	RCBA-UMU
Phragmatobia fuliginosa	IBLAO1034-14	AOC Lep 01129	658	BOLD:AAA6178	KY370727	Spain	RCBA-UMU
Phragmatobia luctifera	FGMLB646-13	BC ZSM Lep 72282	626	BOLD:AAL4874	KY370545	Germany	ZSM
Rhyparia purpurata	IBLAO836-12	AOC Lep 00931	658	BOLD:AAD5293	KY370634	Spain	RCBA-UMU
Setina cantabrica	IBLAO822-12	AOC Lep 00917	658	BOLD:ABZ1427	On progress	Spain	RCBA-UMU
Setina flavicans	IBLAO534-12	AOC Lep 00629	658	BOLD:AAC0260	On progress	Spain	RCBA-UMU
Setina flavicans	IBLAO535-12	AOC Lep 00630	658	BOLD:AAC0260	On progress	Spain	RCBA-UMU
Spilosoma lubricipeda	IBLAO975-14	AOC Lep 01070	658	BOLD:AAB2233	KY370746	Spain	RCBA-UMU
Spilosoma lubricipeda	IBLAO976-14	AOC Lep 01071	658	BOLD:AAB2233	KY370547	Spain	RCBA-UMU
Spilosoma lutea	IBLAO973-14	AOC Lep 01068	658	BOLD:AAC0134	KY370592	Spain	RCBA-UMU
Spilosoma urticae	GWOSA795-10	BC ZSM Lep 36661	612	BOLD:AAE9166	HQ565498	Germany	ZSM
Thumatha senex	GWOR4212-09	BC ZSM Lep 21496	658	BOLD:AAC9508	JF415442	Germany	ZSM
Thumatha senex	GWORK491-09	BC ZSM Lep 21821	658	BOLD:AAC9508	JF415441	Germany	ZSM
Tyria jacobaeae	IBLAO837-12	AOC Lep 00932	658	BOLD:AAB5189	KY370645	Spain	RCBA-UMU
Tyria jacobaeae	IBLAO870-12	AOC Lep 00965	658	BOLD:AAB5189	KY370744	Spain	RCBA-UMU
Utetheisa pulchella	IBLAO558-12	AOC Lep 00653	658	BOLD:AAF0098	KY370609	Spain	RCBA-UMU
Utetheisa pulchella	IBLAO559-12	AOC Lep 00654	658	BOLD:AAF0098	KY370652	Spain	RCBA-UMU
Watsonarctia deserta	IBLAO540-12	AOC Lep 00635	658	BOLD:ABW8791	KY370729	Spain	RCBA-UMU
Watsonarctia deserta	IBLAO541-12	AOC Lep 00636	658	BOLD:ABW8791	KY370709	Spain	RCBA-UMU
**Boletobiinae**							
Parascotia fuliginaria	IBLAO762-12	AOC Lep 00857	658	BOLD:AAC4237	KJ379043	Spain	RCBA-UMU
Parascotia fuliginaria	IBLAO877-12	AOC Lep 00972	658	BOLD:AAC4237	KJ377890	Spain	RCBA-UMU
Parascotia nisseni	IBLAO765-12	AOC Lep 00860	658	BOLD:AAP7326	KY370695	Spain	RCBA-UMU
Parascotia nisseni	IBLAO766-12	AOC Lep 00861	658	BOLD:AAP7326	KY370674	Spain	RCBA-UMU
**Calpinae**							
Calyptra thalictri	LEATH585-14	TLMF Lep 15797	658	BOLD:AAA7764	KY370628	Italy	NST

Identification	Process ID	Sample ID	COI-5P Seq. Length	BIN	Genbank	Country	Institution
Calyptra thalictri	LEATH586-14	TLMF Lep 15798	658	BOLD:AAA7764	KY370675	Italy	NST
Calyptra thalictri	PHLAF258-11	TLMF Lep 05428	658	BOLD:AAA7764	KY370731	Macedonia	TLF
**Erebinae**							
Apopestes spectrum	IBLAO1133-14	AOC Lep 01228	658	BOLD:AAJ1868	KY370691	Spain	RCBA-UMU
Araeopteron ecphaea	GWOTA905-13	BC ZSM Lep 75106	658	BOLD:ACJ7072	KY370739	Italy	ZSM
Autophila cataphanes	IBLAO936-12	AOC Lep 01031	658	BOLD:AAO0894	KY370542	Spain	RCBA-UMU
Autophila dilucida	IBLAO037-11	AOC Lep 00132	658	BOLD:AAL6832	KY370667	Spain	RCBA-UMU
Autophila dilucida	IBLAO038-11	AOC Lep 00133	658	BOLD:AAL6832	KY370604	Spain	RCBA-UMU
Callistege mi	IBLAO815-12	AOC Lep 00910	658	BOLD:AAD6424	KY370690	Spain	RCBA-UMU
Callistege mi	IBLAO816-12	AOC Lep 00911	658	BOLD:AAD6424	KY370638	Spain	RCBA-UMU
Catephia alchymista	IBLAO343-12	AOC Lep 00438	658	BOLD:AAK2247	KY370569	Spain	RCBA-UMU
Catephia alchymista	IBLAO1032-14	AOC Lep 01127	658	BOLD:AAK2247	KY370721	Spain	RCBA-UMU
Catocala conjuncta	IBLAO490-12	AOC Lep 00585	658	BOLD:AAK2298	KY370605	Spain	RCBA-UMU
Catocala conjuncta	IBLAO491-12	AOC Lep 00586	658	BOLD:AAK2298	KY370631	Spain	RCBA-UMU
Catocala conversa	GWORR860-10	AOC Lep 00005	658	BOLD:AAE4407	JN263638	Spain	RCBA-UMU
Catocala conversa	GWORR861-10	AOC Lep 00006	658	BOLD:AAE4407	JN263639	Spain	RCBA-UMU
Catocala conversa	IBLAO340-12	AOC Lep 00435	658	BOLD:AAE4407	KY370724	Spain	RCBA-UMU
Catocala dilecta	IBLAO492-12	AOC Lep 00587	541	BOLD:AAI7371	KY370597	Spain	RCBA-UMU
Catocala dilecta	IBLAO956-14	AOC Lep 01051	658	BOLD:AAI7371	KY370696	Spain	RCBA-UMU
Catocala electa	GWORE2045-09	BC ZSM Lep 22443	658	BOLD:AAD1952	GU654959	Germany	ZSM
Catocala electa	GWORO769-09	BC ZSM Lep 30461	658	BOLD:AAD1952	GU688493	Germany	ZSM
Catocala elocata	IBLAO194-11	AOC Lep 00289	658	BOLD:ABZ5495	KY370632	Spain	RCBA-UMU
Catocala elocata	IBLAO1030-14	AOC Lep 01125	658	BOLD:ABZ5495	KY370578	Spain	RCBA-UMU
Catocala fraxini	IBLAO1084-14	AOC Lep 01179	658	BOLD:AAC6489	KY370611	Spain	RCBA-UMU
Catocala fulminea	GWORL406-09	BC ZSM Lep 22308	658	BOLD:AAB9908	GU686884	Germany	ZSM
Catocala fulminea	FBLMV292-09	BC ZSM Lep 28272	658	BOLD:AAB9908	GU707342	Germany	RCTG
Catocala fulminea	GWORO766-09	BC ZSM Lep 30458	620	BOLD:AAB9908	GU688494	Germany	ZSM
Catocala fulminea	GWORO767-09	BC ZSM Lep 30459	658	BOLD:AAB9908	HQ957500	Germany	ZSM
Catocala mariana	IBLAO044-11	AOC Lep 00139	658	BOLD:AAF2515	KY370689	Spain	RCBA-UMU
Catocala mariana	IBLAO1028-14	AOC Lep 01123	658	BOLD:AAF2515	KY370751	Spain	RCBA-UMU
Catocala nupta	IBLAO753-12	AOC Lep 00848	658	BOLD:ACE8723	KY370570	Spain	RCBA-UMU
Catocala nupta	IBLAO1088-14	AOC Lep 01183	658	BOLD:ACE8723	KY370666	Spain	RCBA-UMU
Catocala nymphaea	IBLAO207-11	AOC Lep 00302	658	BOLD:AAE9291	KY370546	Spain	RCBA-UMU
Catocala nymphaea	IBLAO954-14	AOC Lep 01049	658	BOLD:AAE9291	KY370576	Spain	RCBA-UMU
Catocala nymphagoga	GWORR862-10	AOC Lep 00007	658	BOLD:ACE3559	HM904603	Spain	RCBA-UMU
Catocala nymphagoga	GWORR863-10	AOC Lep 00008	658	BOLD:ACE3559	JN263640	Spain	RCBA-UMU
Catocala optata	IBLAO787-12	AOC Lep 00882	658	BOLD:AAE9294	KY370687	Spain	RCBA-UMU
Catocala optata	IBLAO1087-14	AOC Lep 01182	658	BOLD:AAE9294	KY370579	Spain	RCBA-UMU
Catocala promissa	IBLAO879-12	AOC Lep 00974	658	BOLD:AAD6782	KY370707	Spain	RCBA-UMU
Catocala promissa	IBLAO1086-14	AOC Lep 01181	658	BOLD:AAD6782	KY370601	Spain	RCBA-UMU
Catocala puerpera	GWOSA337-10	BC ZSM Lep 31073	658	BOLD:AAI7405	HQ565290	Italy	ZSM
Catocala sponsa	IBLAO1089-14	AOC Lep 01184	658	BOLD:AAD6876	KY370740	Spain	RCBA-UMU
Clytie illunaris	IBLAO410-12	AOC Lep 00505	658	BOLD:AAK5589	KY370622	Spain	RCBA-UMU

Identification	Process ID	Sample ID	COI-5P Seq. Length	BIN	Genbank	Country	Institution
Clytie illunaris	IBLAO411-12	AOC Lep 00506	658	BOLD:AAK5589	KY370722	Spain	RCBA-UMU
Drasteria cailino	IBLAO342-12	AOC Lep 00437	658	BOLD:AAN4277	KY370737	Spain	RCBA-UMU
Drasteria cailino	IBLAO356-12	AOC Lep 00451	658	BOLD:AAN4277	KY370543	Spain	RCBA-UMU
Dysgonia algira	IBLAO361-12	AOC Lep 00456	658	BOLD:AAI8867	KY370637	Spain	RCBA-UMU
Dysgonia algira	IBLAO362-12	AOC Lep 00457	658	BOLD:AAI8867	KY370693	Spain	RCBA-UMU
Euclidia glyphica	IBLAO811-12	AOC Lep 00906	658	BOLD:AAD0810	KY370665	Spain	RCBA-UMU
Euclidia glyphica	IBLAO838-12	AOC Lep 00933	658	BOLD:AAD0810	KY370742	Spain	RCBA-UMU
Grammodes bifasciata	GWOSA372-10	BC ZSM Lep 31108	639	BOLD:AAN0893	HQ565324	Italy	ZSM
Grammodes stolida	GWOSK886-11	BC ZSM Lep 49387	658	BOLD:AAG5842	JN263667	Cyprus	ZSM
Lygephila craccae	IBLAO178-11	AOC Lep 00273	658	BOLD:AAY4991	KY370617	Spain	RCBA-UMU
Lygephila fonti	IBLAO750-12	AOC Lep 00845	658	BOLD:ABW9402	KY370706	Spain	RCBA-UMU
Lygephila lusoria	IBLAO210-11	AOC Lep 00305	658	BOLD:AAY7719	KY370639	Spain	RCBA-UMU
Lygephila lusoria	IBLAO262-11	AOC Lep 00357	658	BOLD:AAY7719	KY370648	Spain	RCBA-UMU
Lygephila pastinum	GWOR3932-09	BC ZSM Lep 21216	658	BOLD:AAC8970	JF415802	Germany	ZSM
Lygephila pastinum	GWOR3936-09	BC ZSM Lep 21220	658	BOLD:AAC8970	JF415803	Germany	ZSM
Lygephila pastinum	GWORL394-09	BC ZSM Lep 22296	646	BOLD:AAC8970	HM903323	Germany	ZSM
Lygephila pastinum	FBLMX277-11	BC ZSM Lep 50488	658	BOLD:AAC8970	KX040741	Germany	ZSM
Minucia lunaris	IBLAO943-12	AOC Lep 01038	658	BOLD:AAF5900	KY370718	Spain	RCBA-UMU
Minucia lunaris	IBLAO1029-14	AOC Lep 01124	658	BOLD:AAF5900	KY370663	Spain	RCBA-UMU
Ophiusa tirhaca	IBLAO969-14	AOC Lep 01064	658	BOLD:ABZ7648	KY370613	Spain	RCBA-UMU
Ophiusa tirhaca	IBLAO970-14	AOC Lep 01065	658	BOLD:ABZ7648	KY370673	Spain	RCBA-UMU
Pandesma robusta	IBLAO941-12	AOC Lep 01036	658	BOLD:AAG5845	KY370551	Spain	RCBA-UMU
Pandesma robusta	IBLAO1031-14	AOC Lep 01126	658	BOLD:AAG5845	KY370624	Spain	RCBA-UMU
Tathorhynchus exsiccata	GWOSD346-10	BC ZSM Lep 40107	658	BOLD:AAD0612	KY370688	Ethiopia	ZSM
Zethes insularis	IBLAO880-12	AOC Lep 00975	658	BOLD:AAJ9303	KY370752	Spain	RCBA-UMU
Zethes insularis	IBLAO881-12	AOC Lep 00976	658	BOLD:AAJ9303	KY370649	Spain	RCBA-UMU
**Eublemminae**							
Eublemma amoena	IBLAO783-12	AOC Lep 00878	658	BOLD:AAN0634	KY370704	Spain	RCBA-UMU
Eublemma amoena	IBLAO784-12	AOC Lep 00879	658	BOLD:AAN0634	KY370682	Spain	RCBA-UMU
Eublemma candidana	IBLAO288-12	AOC Lep 00383	658	BOLD:AAL4751	KY370568	Spain	RCBA-UMU
Eublemma candidana	IBLAO775-12	AOC Lep 00870	658	BOLD:AAL4751	KY370555	Spain	RCBA-UMU
Eublemma cochylioides	IBLAO074-11	AOC Lep 00169	658	BOLD:ACE8949	KY370660	Spain	RCBA-UMU
Eublemma cochylioides	IBLAO767-12	AOC Lep 00862	658	BOLD:ACE8949	KY370654	Spain	RCBA-UMU
Eublemma ostrina	GWORR872-10	AOC Lep 00017	658	BOLD:AAG1829	HM904606	Spain	RCBA-UMU
Eublemma ostrina	IBLAO084-11	AOC Lep 00179	658	BOLD:AAG1829	KY370599	Spain	RCBA-UMU
Eublemma parva	IBLAO289-12	AOC Lep 00384	658	BOLD:AAM5884	KY370620	Spain	RCBA-UMU
Eublemma parva	IBLAO900-12	AOC Lep 00995	658	BOLD:AAM5884	KY370627	Spain	RCBA-UMU
Eublemma polygramma	IBLAO481-12	AOC Lep 00576	658	BOLD:AAL3781	KY370535	Spain	RCBA-UMU
Eublemma polygramma	IBLAO482-12	AOC Lep 00577	658	BOLD:AAL3781	KY370747	Spain	RCBA-UMU
Eublemma pura	GWORR875-10	AOC Lep 00020	578	BOLD:AAP3973	JF848778	Spain	RCBA-UMU
Eublemma pura	IBLAO902-12	AOC Lep 00997	658	BOLD:AAP3973	KY370668	Spain	RCBA-UMU
Eublemma purpurina	IBLAO738-12	AOC Lep 00833	658	BOLD:AAE4672	KY370661	Spain	RCBA-UMU
Eublemma purpurina	IBLAO1027-14	AOC Lep 01122	658	BOLD:AAE4672	KY370671	Spain	RCBA-UMU

Identification	Process ID	Sample ID	COI-5P Seq. Length	BIN	Genbank	Country	Institution
Eublemma rietzi	GWOSH485-10	BC ZSM Lep 40246	658	BOLD:AAQ3856	JF851463	Spain	ZSM
Eublemma rosea	GWOSH484-10	BC ZSM Lep 40245	658	BOLD:AAQ3856	JF851462	Russia	ZSM
Eublemma scitula	IBLAO899-12	AOC Lep 00994	646	BOLD:ACD0717	KY370571	Spain	RCBA-UMU
Eublemma scitula	IBLAO901-12	AOC Lep 00996	646	BOLD:ACD0717	KY370712	Spain	RCBA-UMU
Metachrostis dardouini	LEATD611-13	TLMF Lep 13258	646	BOLD:ACK1973	KY370730	Italy	TLF
Metachrostis dardouini	LEATD614-13	TLMF Lep 13261	646	BOLD:ACK1973	KY370583	Italy	NST
Metachrostis velox	IBLAO904-12	AOC Lep 00999	646	BOLD:AAH6930	KY370553	Spain	RCBA-UMU
Metachrostis velox	IBLAO937-12	AOC Lep 01032	646	BOLD:AAH6930	KY370572	Spain	RCBA-UMU
Odice blandula	IBLAO199-11	AOC Lep 00294	658	BOLD:AAY5665	KY370680	Spain	RCBA-UMU
Odice blandula	IBLAO200-11	AOC Lep 00295	658	BOLD:AAY5665	KY370651	Spain	RCBA-UMU
Odice jucunda	IBLAO484-12	AOC Lep 00579	658	BOLD:AAZ6317	KY370723	Spain	RCBA-UMU
Odice jucunda	IBLAO771-12	AOC Lep 00866	658	BOLD:AAZ6317	KY370701	Spain	RCBA-UMU
Odice pergrata	IBLAO785-12	AOC Lep 00880	658	BOLD:ACD0937	KY370607	Spain	RCBA-UMU
Odice pergrata	IBLAO1026-14	AOC Lep 01121	658	BOLD:ACD0937	KY370686	Spain	RCBA-UMU
Odice suava	LENOA664-11	LN-BD0664	307		KY370635	France	RCBD
Odice suava	LENOA665-11	LN-BD0665	307		KY370749	France	RCBD
Rhypagla lacernaria	IBLAO173-11	AOC Lep 00268	658	BOLD:AAY8463	KY370683	Spain	RCBA-UMU
Rhypagla lacernaria	IBLAO744-12	AOC Lep 00839	658	BOLD:AAY8463	KY370748	Spain	RCBA-UMU
**Phytometrinae**							
Colobochyla salicalis	GWORO435-09	BC ZSM Lep 19790	658	BOLD:AAD4105	GU688242	Germany	ZSM
Colobochyla salicalis	GWORK470-09	BC ZSM Lep 21800	648	BOLD:AAD4105	JF415763	Germany	ZSM
Colobochyla salicalis	FBLMV258-09	BC ZSM Lep 28238	658	BOLD:AAD4105	JF415762	Germany	ZSM
Phytometra sanctiflorentis	GWORR866-10	AOC Lep 00011	623	BOLD:AAL4238	HM904605	Spain	RCBA-UMU
Phytometra sanctiflorentis	GWORR867-10	AOC Lep 00012	658	BOLD:AAL4238	JN276208	Spain	RCBA-UMU
Phytometra sanctiflorentis	IBLAO934-12	AOC Lep 01029	658	BOLD:AAL4238	KY370705	Spain	RCBA-UMU
Phytometra sanctiflorentis	IBLAO967-14	AOC Lep 01062	658	BOLD:AAL4238	KY370662	Spain	RCBA-UMU
Phytometra sanctiflorentis	IBLAO968-14	AOC Lep 01063	658	BOLD:AAL4238	KY370560	Spain	RCBA-UMU
Phytometra viridaria	IBLAO423-12	AOC Lep 00518	658	BOLD:AAD4078	KY370738	Spain	RCBA-UMU
Phytometra viridaria	IBLAO424-12	AOC Lep 00519	658	BOLD:AAD4078	KY370536	Spain	RCBA-UMU
Raparna conicephala	IBLAO081-11	AOC Lep 00176	658	BOLD:AAG3109	KY370719	Spain	RCBA-UMU
Raparna conicephala	IBLAO214-11	AOC Lep 00309	586	BOLD:AAG3109	KY370659	Spain	RCBA-UMU
**Aventiinae**							
Laspeyria flexula	FBLMV256-09	BC ZSM Lep 28236	658	BOLD:AAC1014	JF415800	Germany	ZSM
Laspeyria flexula	GWOSC417-10	BC ZSM Lep 36093	658	BOLD:AAC1014	HQ566463	Germany	ZSM
Laspeyria flexula	GWOSC418-10	BC ZSM Lep 36094	658	BOLD:AAC1014	HQ566464	Germany	ZSM
Trisateles emortualis	GWORK471-09	BC ZSM Lep 21801	656	BOLD:AAC2587	JF415844	Germany	ZSM
Trisateles emortualis	GWORL451-09	BC ZSM Lep 22353	658	BOLD:AAC2587	GU686852	Germany	ZSM
Trisateles emortualis	FBLMT432-09	BC ZSM Lep 24992	658	BOLD:AAC2587	GU654965	Germany	RCTG
**Herminiinae**							
Herminia grisealis	FBLMV271-09	BC ZSM Lep 28251	633	BOLD:AAC3337	JF415785	Germany	ZSM
Herminia grisealis	FBLMV273-09	BC ZSM Lep 28253	603	BOLD:AAC3337	JF415786	Germany	ZSM
Herminia grisealis	FBLMV423-09	BC ZSM Lep 28403	658	BOLD:AAC3337	GU707372	Germany	RCTG
Herminia grisealis	FBLMW103-10	BC ZSM Lep 29033	658	BOLD:AAC3337	HQ563388	Germany	RCAH

Identification	Process ID	Sample ID	COI-5P Seq. Length	BIN	Genbank	Country	Institution
Herminia tarsicrinalis	FBLMV269-09	BC ZSM Lep 28249	658	BOLD:AAC1537	JF415788	Germany	ZSM
Herminia tarsicrinalis	FBLMV270-09	BC ZSM Lep 28250	658	BOLD:AAC1537	JF415787	Germany	ZSM
Herminia tarsipennalis	IBLAO963-14	AOC Lep 01058	658	BOLD:AAC1538	KY370677	Spain	RCBA-UMU
Herminia tarsipennalis	IBLAO964-14	AOC Lep 01059	658	BOLD:AAC1538	KY370587	Spain	RCBA-UMU
Idia calvaria	PHLAC497-10	TLMF Lep 02532	634	BOLD:AAK4189	JF860069	Italy	TLF
Idia calvaria	LEATH813-14	TLMF Lep 16025	658	BOLD:AAK4189	KY370612	Italy	TLF
Macrochilo cribrumalis	FBLMV262-09	BC ZSM Lep 28242	658	BOLD:AAC6065	JF415806	Germany	ZSM
Macrochilo cribrumalis	FBLMV263-09	BC ZSM Lep 28243	658	BOLD:AAC6065	JF415805	Germany	ZSM
Nodaria nodosalis	IBLAO075-11	AOC Lep 00170	658	BOLD:AAK3749	KY370725	Spain	RCBA-UMU
Nodaria nodosalis	IBLAO076-11	AOC Lep 00171	658	BOLD:AAK3749	KY370735	Spain	RCBA-UMU
Paracolax tristalis	IBLAO960-14	AOC Lep 01055	658	BOLD:AAE6800	KY370708	Spain	RCBA-UMU
Paracolax tristalis	IBLAO1108-14	AOC Lep 01203	658	BOLD:AAE6800	KY370594	Spain	RCBA-UMU
Pechipogo plumigeralis	IBLAO485-12	AOC Lep 00580	658	BOLD:AAI4196	KY370565	Spain	RCBA-UMU
Pechipogo plumigeralis	IBLAO486-12	AOC Lep 00581	658	BOLD:AAI4196	KY370564	Spain	RCBA-UMU
Pechipogo strigilata	GWOR3866-09	BC ZSM Lep 21150	658	BOLD:AAE7062	JF415832	Germany	ZSM
Pechipogo strigilata	FBLMU116-09	BC ZSM Lep 25626	658	BOLD:AAE7062	GU654977	Germany	RCTG
Pechipogo strigilata	FBLMV259-09	BC ZSM Lep 28239	658	BOLD:AAE7062	JF415831	Germany	ZSM
Pechipogo strigilata	FBLMV260-09	BC ZSM Lep 28240	658	BOLD:AAE7062	JF415830	Germany	ZSM
Pechipogo strigilata	FBLMV261-09	BC ZSM Lep 28241	658	BOLD:AAE7062	JF415829	Germany	ZSM
Polypogon tentacularia	IBLAO475-12	AOC Lep 00570	658	BOLD:AAF0435	KY370598	Spain	RCBA-UMU
Zanclognatha lunalis	IBLAO961-14	AOC Lep 01056	658	BOLD:AAG5877	KY370621	Spain	RCBA-UMU
Zanclognatha lunalis	IBLAO962-14	AOC Lep 01057	632	BOLD:AAG5877	KY370646	Spain	RCBA-UMU
Zanclognatha zelleralis	FBLMZ544-12	BC ZSM Lep 61300	658	BOLD:AAJ9773	KX044346	Germany	RCPL
**Hypeninae**							
Hypena crassalis	IBLAO966-14	AOC Lep 01061	658	BOLD:AAD1366	KY370615	Spain	RCBA-UMU
Hypena lividalis	IBLAO897-12	AOC Lep 00992	658	BOLD:AAE1121	KY370567	Spain	RCBA-UMU
Hypena lividalis	IBLAO948-12	AOC Lep 01043	658	BOLD:AAE1121	KY370600	Spain	RCBA-UMU
Hypena obesalis	GWORK494-09	BC ZSM Lep 21824	658	BOLD:AAF3765	JF415791	Germany	ZSM
Hypena obesalis	GWORK504-09	BC ZSM Lep 21834	658	BOLD:AAF3765	JF415790	Germany	ZSM
Hypena obesalis	GWORL312-09	BC ZSM Lep 22024	658	BOLD:AAF3765	GU686956	Germany	ZSM
Hypena obsitalis	IBLAO938-12	AOC Lep 01033	658	BOLD:AAK3686	KY370623	Spain	RCBA-UMU
Hypena proboscidalis	IBLAO443-12	AOC Lep 00538	658	BOLD:AAB6485	KY370538	Spain	RCBA-UMU
Hypena proboscidalis	IBLAO1025-14	AOC Lep 01120	658	BOLD:AAB6485	KY370588	Spain	RCBA-UMU
Hypena rostralis	GWOR3929-09	BC ZSM Lep 21213	658	BOLD:AAD2713	JF415796	Germany	ZSM
Hypena rostralis	GWORL397-09	BC ZSM Lep 22299	658	BOLD:AAD2713	GU686892	Germany	ZSM
Hypena rostralis	GWORL448-09	BC ZSM Lep 22350	658	BOLD:AAD2713	GU686849	Germany	ZSM
Hypena rostralis	GWORE1978-09	BC ZSM Lep 22376	658	BOLD:AAD2713	GU654962	Germany	ZSM
Hypena rostralis	FBLMV277-09	BC ZSM Lep 28257	658	BOLD:AAD2713	JF415795	Germany	ZSM
**Hypenodinae**							
Schrankia costaestrigalis	GWOSI565-10	BC ZSM Lep 44411	658	BOLD:AAD1543	KX040235	Germany	ZSM
Schrankia costaestrigalis	GWOSI799-10	BC ZSM Lep 49205	658	BOLD:AAD1543	JN273701	Germany	ZSM
Schrankia costaestrigalis	FBLMZ641-12	BC ZSM Lep 61492	658	BOLD:AAD1543	KX045756	Germany	RCPL
Schrankia costaestrigalis	GBLAB695-13	BC ZSM Lep 75466	658	BOLD:AAD1543	KY370585	Germany	ZSM

Identification	Process ID	Sample ID	COI-5P Seq. Length	BIN	Genbank	Country	Institution
**Lymantriinae**							
Albarracina warionis	IBLAO946-12	AOC Lep 01041	658	BOLD:ACD0639	KY370734	Spain	RCBA-UMU
Arctornis l-nigrum	IBLAO977-14	AOC Lep 01072	658	BOLD:AAD9740	KY370633	Spain	RCBA-UMU
Arctornis l-nigrum	IBLAO1085-14	AOC Lep 01180	658	BOLD:AAD9740	KY370636	Spain	RCBA-UMU
Calliteara pudibunda	IBLAO981-14	AOC Lep 01076	658	BOLD:AAB5204	KY370608	Spain	RCBA-UMU
Calliteara pudibunda	IBLAO983-14	AOC Lep 01078	658	BOLD:AAB5204	KY370591	Spain	RCBA-UMU
Dicallomera fascelina	IBLAO982-14	AOC Lep 01077	658	BOLD:AAE7512	KY370625	Spain	RCBA-UMU
Euproctis chrysorrhoea	IBLAO564-12	AOC Lep 00659	658	BOLD:ABY5435	KY370643	Spain	RCBA-UMU
Euproctis chrysorrhoea	IBLAO565-12	AOC Lep 00660	658	BOLD:ABY5435	KY370676	Spain	RCBA-UMU
Euproctis similis	IBLAO979-14	AOC Lep 01074	658	BOLD:AAC3712	KY370580	Spain	RCBA-UMU
Euproctis similis	IBLAO980-14	AOC Lep 01075	658	BOLD:AAC3712	KY370692	Spain	RCBA-UMU
Laelia coenosa	GWOSQ517-11	BC ZSM Lep 56998	656	BOLD:AAZ9969	KX045456	Hungary	RCCZ
Leucoma salicis	IBLAO562-12	AOC Lep 00657	658	BOLD:AAA5528	KY370650	Spain	RCBA-UMU
Leucoma salicis	IBLAO563-12	AOC Lep 00658	658	BOLD:AAA5528	KY370647	Spain	RCBA-UMU
Lymantria atlantica	IBLAO561-12	AOC Lep 00656	658	BOLD:AAM9785	KY370556	Spain	RCBA-UMU
Lymantria atlantica	IBLAO1120-14	AOC Lep 01215	658	BOLD:AAM9785	KY370562	Spain	RCBA-UMU
Lymantria dispar	IBLAO560-12	AOC Lep 00655	658	BOLD:AAA2052	KY370641	Spain	RCBA-UMU
Lymantria dispar	IBLAO770-12	AOC Lep 00865	658	BOLD:AAA2052	KY370716	Spain	RCBA-UMU
Lymantria monacha	IBLAO978-14	AOC Lep 01073	658	BOLD:AAA5537	KY370629	Spain	RCBA-UMU
Lymantria monacha	IBLAO1137-14	AOC Lep 01232	658	BOLD:AAA5537	KY370733	Spain	RCBA-UMU
Ocneria rubea	IBLAO566-12	AOC Lep 00661	629	BOLD:AAZ8646	KY370559	Spain	RCBA-UMU
Ocneria rubea	IBLAO567-12	AOC Lep 00662	658	BOLD:AAZ8646	KY370726	Spain	RCBA-UMU
Ocneria rubea	IBLAO1035-14	AOC Lep 01130	658	BOLD:AAZ8646	KY370640	Spain	RCBA-UMU
Orgyia antiqua	IBLAO1096-14	AOC Lep 01191	658	BOLD:AAA6432	KY370575	Spain	RCBA-UMU
Orgyia antiqua	IBLAO1097-14	AOC Lep 01192	658	BOLD:AAA6432	KY370586	Spain	RCBA-UMU
Orgyia aurolimbata	IBLAO1098-14	AOC Lep 01193	658	BOLD:ACL5700	KY370714	Spain	RCBA-UMU
Orgyia dubia	IBLAO1095-14	AOC Lep 01190	658	BOLD:ACL5770	KY370732	Spain	RCBA-UMU
Orgyia dubia	IBLAO1139-14	AOC Lep 01234	658	BOLD:ACL5732	KY370642	Spain	RCBA-UMU
Orgyia recens	LEATG202-14	TLMF Lep 13989	634	BOLD:AAK5492	KY370710	Italy	TLF
Orgyia trigotephras	IBLAO570-12	AOC Lep 00665	658	BOLD:ABW9167	KY370656	Spain	RCBA-UMU
Orgyia trigotephras	IBLAO571-12	AOC Lep 00666	658	BOLD:ABW9167	KY370664	Spain	RCBA-UMU
**Rivulinae**							
Rivula sericealis	IBLAO1129-14	AOC Lep 01224	614	BOLD:AAB4777	KY370540	Spain	RCBA-UMU
Zebeeba falsalis	GWORR864-10	AOC Lep 00009	658	BOLD:AAJ9181	HM904604	Spain	RCBA-UMU
Zebeeba falsalis	GWORR865-10	AOC Lep 00010	658	BOLD:AAJ9181	KY370603	Spain	RCBA-UMU
**Scoliopteryginae**							
Scoliopteryx libatrix	IBLAO487-12	AOC Lep 00582	658	BOLD:ACE7197	KY370544	Spain	RCBA-UMU
Scoliopteryx libatrix	IBLAO869-12	AOC Lep 00964	658	BOLD:ACE7197	KY370702	Spain	RCBA-UMU
