# Supplementary material for: Close congruence between Barcode Index Numbers (bins) and species boundaries in the Erebidae (Lepidoptera: Noctuoidea) of the Iberian Peninsula
Source: Biodivers Data J. 2017 Aug 8;(5):e19840. doi: 10.3897/BDJ.5.e19840 (PMC5558050; doi:10.3897/BDJ.5.e19840)
Supplement: Supplementary material 2 — Systematic list of subfamilies and species, barcode gap analysis (Mean and Maximum intraspecific variation and distance to nearest neighbor NN) for 160 Iberian species in the all European Erebidae [file bdj-05-e19840-s002.pdf]

Appendix S2: List of subfamilies and species, barcode gap analysis (intraspecific variation and distance to nearest neighbor) for 160 Iberian species in the all Erebidae European

Species	Mean Intra-Sp	Max Intra-Sp	Nearest Neighbour	Nearest Species	Distance to NN
**Scoliopteryginae**					
Scoliopteryx libatrix (Linnaeus, 1758)	0	0	PHLAC497-10	*Idia calvaria*	8.93
**Rivulinae**					
Rivula sericealis (Scopoli, 1763)	N/A	N/A	IBLAO1105-14	*Euplagia quadripunctaria*	8.65
Zebeeba falsalis (Herrich-Schäffer, 1839)	0	0	IBLAO857-12	*Parasemia plantaginis*	10.14
**Hypeninae**					
Hypena (Hypena) proboscidalis (Linnaeus, 1758)	0	0	GWORL312-09	*Hypena obesalis*	7.26
Hypena (Hypena) rostralis (Linnaeus, 1758)	0	0	GWORL312-09	*Hypena obesalis*	4.58
Hypena (Hypena) obesalis Treitschke, 1829	0.2	0.31	GWORL397-09	*Hypena rostralis*	4.58
Hypena (Hypena) obsitalis (Hübner, 1813)	N/A	N/A	GWORL312-09	*Hypena obesalis*	5.43
Hypena (Hypena) lividalis (Hübner, 1796)	0.15	0.15	IBLAO938-12	*Hypena obsitalis*	8.28
Hypena (Hypena) crassalis (Fabricius, 1787)	N/A	N/A	GWORL312-09	*Hypena obesalis*	5.74
**Lymantriinae**					
Arctornis l-nigrum (Müller, 1764)	0	0	IBLAO982-14	*Dicallomera fascelina*	10.16
Leucoma salicis (Linnaeus, 1758)	0.62	0.62	IBLAO872-12	*Coscinia striata*	12.84
Lymantria dispar (Linnaeus, 1758)	0.31	0.31	IBLAO978-14	*Lymantria monacha*	10.67
Lymantria monacha (Linnaeus, 1758)	0	0	IBLAO1105-14	*Euplagia quadripunctaria*	10.14
Lymantria atlantica (Rambur, 1837)	0.15	0.15	IBLAO511-12	*Miltochrista miniata*	12.47
Ocneria rubea (Denis & Schiffermüller, 1775)	0.1	0.16	FBLMV263-09	*Macrochilo cribrumalis*	11.39
Albarracina warionis (Oberthür, 1881)	N/A	N/A	FBLMV261-09	*Pechipogo strigilata*	7.89
Euproctis (Euproctis) chrysorrhoea (Linnaeus, 1758)	0	0	IBLAO560-12	*Lymantria dispar*	12.82
Euproctis (Sphrageidus) similis (Fuessly, 1775)	0.15	0.15	IBLAO538-12	*Diacrisia sannio*	14.74
Laelia coenosa (Hübner, 1808)	N/A	N/A	IBLAO857-12	*Parasemia plantaginis*	10.88
Calliteara pudibunda (Linnaeus, 1758)	0.31	0.31	FGMLB646-13	*Phragmatobia luctifera*	10.38
Dicallomera fascelina (Linnaeus, 1758)	N/A	N/A	IBLAO1085-14	*Arctornis l-nigrum*	10.16
Orgyia (Clethrogyna) recens (Hübner 1819)	N/A	N/A	FBLMV273-09	*Herminia grisealis*	11.35
Orgyia (Clethrogyna) aurolimbata Guenée, 1835	N/A	N/A	IBLAO199-11	*Odice blandula*	11.93
Orgyia (Clethrogyna) dubia (Tauscher, 1806)	2.35	2.35	IBLAO1098-14	*Orgyia aurolimbata*	13.48
Orgyia (Clethrogyna) trigotephras Boisduval, 1829	0.46	0.46	IBLAO522-12	*Eilema lurideola*	12.49
Orgyia (Orgyia) antiqua (Linnaeus, 1758)	0	0	IBLAO816-12	*Callistege mi*	10.16
**Arctiinae**					
Miltochrista miniata (Forster, 1771)	0	0	IBLAO475-12	*Polypogon tentacularia*	9.44
Nudaria mundana (Linnaeus, 1761)	N/A	N/A	IBLAO475-12	*Polypogon tentacularia*	9.27
Thumatha senex (Hübner, 1708)	0.15	0.15	IBLAO475-12	*Polypogon tentacularia*	9.44
Paidia rica (Freyer, 1858)	0	0	FBLMZ544-12	*Zanclognatha zelleralis*	7.89
Cybosia mesomella (Linnaeus, 1758)	0	0	IBLAO524-12	*Eilema lutarella*	7.97
Pelosia muscerda (Hufnagel, 1766)	0.13	0.2	GBLAA1109-15	*Pelosia obtusa*	7.01
Pelosia obtusa (Herrich-Schäffer, 1852)	N/A	N/A	IBLAO887-12	*Eilema predotae*	6.73
Lithosia quadra (Linnaeus, 1758)	0	0	IBLAO887-12	*Eilema predotae*	7.76
Atolmis rubricollis (Linnaeus, 1758)	0	0	IBLAO887-12	*Eilema predotae*	6.4
Eilema griseola (Hübner, 1803)	0	0	IBLAO524-12	*Eilema lutarella*	6.76
Eilema depressa (Esper, 1787)	0	0	IBLAO887-12	*Eilema predotae*	6.59
Eilema albicosta (Rogenhofer, 1894)	N/A	N/A	IBLAO524-12	*Eilema lutarella*	4.61
Eilema interpositella Strand, 1920	0	0	IBLAO524-12	*Eilema lutarella*	4.1
Eilema uniola (Rambur, 1866)	0.15	0.15	IBLAO524-12	*Eilema lutarella*	4.28
Eilema lutarella (Linnaeus, 1758)	0	0	IBLAO531-12	*Eilema pygmaeola*	3.79
Eilema lurideola (Zincken, 1817)	0	0	IBLAO524-12	*Eilema lutarella*	4.27
Eilema caniola (Hübner, 1808)	0.46	0.46	IBLAO825-12	*Eilema pseudocomplana*	4.93
Eilema palliatella (Scopoli, 1763)	0.46	0.46	IBLAO928-12	*Eilema complana*	4.11

Eilema complana(Linnaeus, 1758)	0.49	1.08	IBLAO825-12	*Eilema pseudocomplana*	3.63
Eilema pseudocomplana (Daniel, 1939)	0.16	0.16	IBLAO928-12	*Eilema complana*	3.63
Eilema marcida (Mann, 1859)	N/A	N/A	IBLAO887-12	*Eilema predotae*	2.19
Eilema pygmaeola (Doubleday, 1847)	0.31	0.31	IBLAO524-12	*Eilema lutarella*	3.79
Eilema predotae (Schawerda, 1927)	N/A	N/A	IBLAO1141-14	*Eilema marcida*	2.19
Eilema rungsi Toulgoet, 1960	N/A	N/A	IBLAO887-12	*Eilema predotae*	6.73
Eilema sororcula (Hufnagel, 1766)	0	0	IBLAO887-12	*Eilema predotae*	5.9
Setina flavicans (Geyer, 1836)	0	0	IBLAO822-12	*Setina cantabrica*	2.34
Setina cantabrica de Freina & Witt, 1985	N/A	N/A	IBLAO534-12	*Setina flavicans*	2.34
Amata phegea (Linnaeus, 1758)	N/A	N/A	FBLMU057-09	*Dysauxes ancilla*	7.62
Dysauxes ancilla (Linnaeus, 1767)	0.16	0.16	LEATG255-14	*Dysauxes punctata*	5.38
Dysauxes punctata (Fabricius, 1781)	0.15	0.15	FBLMU057-09	*Dysauxes ancilla*	5.38
Spilosoma lutea (Hufnagel, 1766)	N/A	N/A	IBLAO790-12	*Diaphora mendica*	7.42
Spilosoma lubricipeda (Linnaeus, 1758)	0	0	IBLAO790-12	*Diaphora mendica*	4.41
Spilosoma urticae (Esper, 1789)	N/A	N/A	FGMLB646-13	*Phragmatobia luctifera*	4.45
Diaphora mendica (Clerck, 1759)	0.15	0.15	IBLAO975-14	*Spilosoma lubricipeda*	4.41
Watsonarctia deserta (Bartel, 1902)	0.15	0.15	FGMLB646-13	*Phragmatobia luctifera*	7.34
Diacrisia sannio (Linnaeus, 1758)	0.61	0.61	IBLAO836-12	*Rhyparia purpurata*	6.72
Rhyparia purpurata (Linnaeus, 1758)	N/A	N/A	IBLAO1033-14	*Diacrisia sannio*	6.72
Ocnogyna zoraida (Graslin, 1837)	N/A	N/A	LNOUB019-10	*Ocnogyna zoraida*	2.35
Ocnogyna baetica (Rambur, 1836)	3.2	3.2	IBLAO790-12	*Diaphora mendica*	5.41
Artimelia latreillei (Godart, 1823)	1.08	1.08	FGMLB646-13	*Phragmatobia luctifera*	6.39
Phragmatobia luctifera (Denis & Schiffermüller, 1775)	N/A	N/A	GWOSA795-10	*Spilosoma urticae*	4.45
Phragmatobia fuliginosa (Linnaeus, 1758)	0.15	0.15	GWOSA795-10	*Spilosoma urticae*	6.78
Parasemia plantaginis (Linnaeus, 1758)	0.46	0.46	IBLAO551-12	*Arctia caja*	5.4
Arctia festiva (Hufnagel, 1766)	0.52	0.52	IBLAO857-12	*Parasemia plantaginis*	5.72
Arctia villica(Linnaeus, 1758)	0.42	2.02	IBLAO0921-12	*Chelis maculosa*	5.14
Arctia caja (Linnaeus, 1758)	0	0	IBLAO857-12	*Parasemia plantaginis*	5.4
Atlantarctia tigrina (Villers, 1789)	0.15	0.15	FGMLB646-13	*Phragmatobia luctifera*	5.52
Hyphoraia testudinaria (Geoffroy, in Fourcroy 1785)	N/A	N/A	IBLAO547-12	*Hyphoraia dejeani*	4.61
Hyphoraia dejeani (Godart, 1822)	0	0	IBLAO546-12	*Hyphoraia testudinaria*	4.61
Chelis maculosa (Gerning, 1780)	0.46	1.3	IBLAO840-12	*Chelis cantabrica*	3.52
Chelis arragonensis (Staudinger, 1894)	0.1	0.16	IBLAO840-12	*Chelis cantabrica*	3.21
Chelis cantabrica Macià, Gastón *et al.*, 2013	0	0	IBLAO842-12	*Chelis arragonensis*	3.21
Callimorpha dominula (Linnaeus, 1758)	0.07	0.17	IBLAO1105-14	*Euplagia quadripunctaria*	4.99
Euplagia quadripunctaria (Poda, 1761)	0	0	FBLMV287-09	*Callimorpha dominula*	4.99
Tyria jacobaeae (Linnaeus, 1758)	0	0	IBLAO1105-14	*Euplagia quadripunctaria*	7.58
Cymbalophora pudica (Esper, 1785)	N/A	N/A	IBLAO1105-14	*Euplagia quadripunctaria*	6.41
Coscinia striata (Linnaeus, 1758)	0	0	IBLAO547-12	*Hyphoraia dejeani*	9.12
Coscinia cribraria (Linnaeus, 1758)	1.08	1.08	IBLAO1105-14	*Euplagia quadripunctaria*	9.64
Utetheisa pulchella (Linnaeus, 1758)	0.31	0.31	IBLAO857-12	*Parasemia plantaginis*	9.09
**Calpinae**					
Calyptra thalictri (Borkhausen, 1790)	0	0	FBLMZ544-12	*Zanclognatha zelleralis*	3.89
**Herminiinae**					
Idia calvaria (Denis et Schiffermüller, 1775)	0	0	FBLMV261-09	*Pechipogo strigilata*	6.55
Paracolax tristalis (Fabricius, 1794)	0	0	FBLMZ544-12	*Zanclognatha zelleralis*	7.39
Nodaria nodosalis (Herrich-Schäffer, 1851)	0.15	0.15	FBLMZ544-12	*Zanclognatha zelleralis*	6.07
Macrochilo cribrumalis (Hübner, 1793)	0	0	IBLAO475-12	*Polypogon tentacularia*	6.56
Zanclognatha zelleralis (Wocke, 1850)	N/A	N/A	IBLAO475-12	*Polypogon tentacularia*	4.42
Zanclognatha lunalis (Scopoli, 1763)	0.16	0.16	FBLMZ544-12	*Zanclognatha zelleralis*	5.12
Pechipogo strigilata (Linnaeus, 1758)	0	0	FBLMZ544-12	*Zanclognatha zelleralis*	4.74
Pechipogo plumigeralis Hübner, 1825	0	0	FBLMV261-09	*Pechipogo strigilata*	4.74
Polypogon tentacularia (Linnaeus, 1758)	N/A	N/A	FBLMZ544-12	*Zanclognatha zelleralis*	4.42
Herminia tarsipennalis (Treitschke, 1835)	0	0	FBLMV261-09	*Pechipogo strigilata*	4.75
Herminia grisealis (Denis & Schiffermüller, 1775)	0	0	FBLMV261-09	*Pechipogo strigilata*	6.26

Herminia tarsicrinalis (Knoch, 1782)	0	0	FBLMV261-09	*Pechipogo strigilata*	7.06
**Hypenodinae**					
Schrankia costaestrigalis (Stephens, 1834)	0.08	0.15	FBLMV287-09	*Callimorpha dominula*	9.41
**Boletobiinae**					
Parascotia nisseni Turati, 1905	0	0	IBLAO877-12	*Parascotia fuliginaria*	6.4
Parascotia fuliginaria (Linnaeus, 1761)	0.62	0.62	IBLAO766-12	*Parascotia nisseni*	6.4
**Phytometrinae**					
Phytometra sanctiflorentis (Boisduval, 1834)	0.06	0.16	IBLAO423-12	*Phytometra viridaria*	4.93
Phytometra viridaria (Clerck, 1759)	0.62	0.62	GWORR867-10	*Phytometra sanctiflorentis*	4.93
Raparna conicephala (Staudinger, 1870)	0.52	0.52	IBLAO934-12	*Phytometra sanctiflorentis*	6.09
Colobochyla salicalis (Denis & Schiffermüller, 1775)	0	0	FBLMZ544-12	*Zanclognatha zelleralis*	8.92
**Aventiinae**					
Laspeyria flexula (Denis & Schiffermüller, 1775)	0.1	0.15	FBLMZ544-12	*Zanclognatha zelleralis*	9.27
Trisateles emortualis (Denis & Schiffermüller, 1775)	0	0	FBLMZ544-12	*Zanclognatha zelleralis*	7.72
**Eublemminae**					
Odice blandula (Rambur, 1858)	0	0	IBLAO785-12	*Odice pergrata*	4.76
Odice pergrata (Rambur, 1858)	0.15	0.15	IBLAO199-11	*Odice blandula*	4.76
Odice jucunda (Hübner, 1813)	0	0	IBLAO744-12	*Rhypagla lacernaria*	8.58
Eublemma candidana Fabricius, 1794	0.15	0.15	IBLAO738-12	*Eublemma purpurina*	7.57
Eublemma parva (Hübner, 1808)	0.31	0.31	IBLAO767-12	*Eublemma cochylioides*	5.41
Eublemma cochylioides (Guenée, 1852)	0	0	IBLAO289-12	*Eublemma parva*	5.41
Eublemma ostrina (Hübner, 1808)	0.15	0.15	GWOSH484-10	*Eublemma rosea*	5.72
Eublemma purpurina (Denis & Schiffermüller, 1775)	0	0	GWOSH484-10	*Eublemma rosea*	5.07
Eublemma rosea (Hübner, 1790)	N/A	N/A	GWOSH485-10	*Eublemma rietzi*	2.02
Eublemma rietzi Yela, Fibiger, Ronkay & Zilli,2010	N/A	N/A	GWOSH484-10	*Eublemma rosea*	2.02
Eublemma amoena (Hübner, 1808)	0	0	IBLAO738-12	*Eublemma purpurina*	6.26
Eublemma pura (Hübner, 1813)	0.17	0.17	IBLAO771-12	*Odice jucunda*	9.46
Eublemma polygramma (Duponchel, 1842)	0	0	IBLAO744-12	*Rhypagla lacernaria*	7.56
Eublemma scitula Rambur, 1833	0	0	IBLAO482-12	*Eublemma polygramma*	10.29
Rhypagla lacernaria (Hübner, 1813)	0.15	0.15	IBLAO482-12	*Eublemma polygramma*	7.56
Metachrostis velox (Hübner, 1813)	0.62	0.62	LEATD614-13	*Metachrostis dardouini*	2.21
Metachrostis dardouini (Boisduval, 1840)	0	0	IBLAO904-12	*Metachrostis velox*	2.21
**Erebinae**					
Araeopteron ecphaea Hampson, 1914	N/A	N/A	FBLMZ544-12	*Zanclognatha zelleralis*	7.89
Catephia alchymista (Denis & Schiffermüller, 1775)	0.15	0.15	IBLAO943-12	*Minucia lunaris*	8.92
Pandesma robusta (Walker, 1858)	0	0	GWOSK886-11	*Grammodes stolida*	7.74
Zethes insularis Rambur, 1833	0.15	0.15	IBLAO970-14	*Ophiusa tirhaca*	7.89
Drasteria cailino (Lefèbvre, 1827)	0.93	0.93	IBLAO410-12	*Clytie illunaris*	7.55
Catocala fulminea (Scopoli, 1763)	0.08	0.16	GWORR861-10	*Catocala conversa*	5.54
Catocala nymphaea (Esper, 1787)	0.46	0.46	GWORR863-10	*Catocala nymphagoga*	5.92
Catocala mariana Rambur, 1858	0	0	IBLAO956-14	*Catocala dilecta*	5.57
Catocala conversa (Esper, 1783)	0	0	IBLAO1084-14	*Catocala fraxini*	4.42
Catocala nymphagoga (Esper, 1787)	0.31	0.31	IBLAO1084-14	*Catocala fraxini*	4.92
Catocala fraxini (Linnaeus, 1758)	N/A	N/A	IBLAO1089-14	*Catocala sponsa*	3.46
Catocala conjuncta (Esper, 1787)	0	0	IBLAO1084-14	*Catocala fraxini*	6.4
Catocala nupta (Linnaeus, 1767)	0	0	IBLAO1089-14	*Catocala sponsa*	4.11
Catocala electa (Vieweg, 1790)	0	0	IBLAO1089-14	*Catocala sponsa*	4.59
Catocala elocata (Esper, 1787)	0	0	IBLAO879-12	*Catocala promissa*	4.42
Catocala puerpera (Girona, 1791)	N/A	N/A	IBLAO194-11	*Catocala elocata*	4.44
Catocala dilecta (Hübner, 1808)	0.19	0.19	IBLAO1089-14	*Catocala sponsa*	3.62
Catocala sponsa (Linnaeus, 1767)	N/A	N/A	IBLAO1084-14	*Catocala fraxini*	3.46

Catocala promissa (Denis & Schiffermüller, 1775)	0	0	IBLAO194-11	*Catocala elocata*	4.42
Catocala optata (Godart, 1824)	0.15	0.15	IBLAO1084-14	*Catocala fraxini*	5.26
Euclidia (Euclidia) glyphica (Linnaeus, 1758)	0	0	IBLAO475-12	*Polypogon tentacularia*	8.41
Euclidia (Callistege) mi (Clerck, 1759)	0	0	FBLMV263-09	*Macrochilo cribrumalis*	9.1
Ophiusa tirhaca (Cramer, 1773)	0	0	IBLAO943-12	*Minucia lunaris*	4.57
Minucia lunaris (Denis & Schiffermüller, 1775)	0.46	0.46	IBLAO410-12	*Clytie illunaris*	3.92
Clytie illunaris (Hübner, 1813)	0	0	IBLAO943-12	*Minucia lunaris*	3.92
Dysgonia algira (Linnaeus, 1767)	0.15	0.15	GWOSA372-10	*Grammodes bifasciata*	7.62
Grammodes bifasciata (Petagna, 1787)	N/A	N/A	GWOSK886-11	*Grammodes stolida*	5.56
Grammodes stolida (Fabricius, 1775)	0.53	2.09	GWOSA372-10	*Grammodes bifasciata*	5.56
Lygephila lusoria (Linnaeus, 1758)	0	0	GWORL394-09	*Lygephila pastinum*	5.85
Lygephila fonti Yela & Calle, 1990	N/A	N/A	GWORL394-09	*Lygephila pastinum*	4.19
Lygephila pastinum (Treitschke, 1826)	0.08	0.16	IBLAO750-12	*Lygephila fonti*	4.19
Lygephila craccae (Denis & Schiffermüller, 1775)	N/A	N/A	GWORL394-09	*Lygephila pastinum*	8.05
Tathorhynchus exsiccata (Lederer, 1855)	0.33	0.96	IBLAO750-12	*Lygephila fonti*	8.25
Autophila dilucida (Hübner, 1808)	0.15	0.15	IBLAO936-12	*Autophila cataphanes*	7.92
Autophila cataphanes (Hübner, 1813)	N/A	N/A	IBLAO038-11	*Autophila dilucida*	7.92
Apopestes spectrum (Esper, 1787)	N/A	N/A	IBLAO210-11	*Lygephila lusoria*	6.72
