# Supplementary material for: Close congruence between Barcode Index Numbers (bins) and species boundaries in the Erebidae (Lepidoptera: Noctuoidea) of the Iberian Peninsula
Source: Biodivers Data J. 2017 Aug 8;(5):e19840. doi: 10.3897/BDJ.5.e19840 (PMC5558050; doi:10.3897/BDJ.5.e19840)
Supplement: Supplementary material 3 — List of 16 Iberian taxa without a BIN assignment [file bdj-05-e19840-s003.pdf]

Appendix S3: List of Iberian Erebidae species without BIN

List of 16 Iberian taxa without a BIN assignment (awaiting DNA barcoding); a species with short sequences are marked with an *.

Subfamily Arctiinae

Pelosia plumosa (Mabille, 1900)
Apaidia mesogona (Godart, 1824)
Zobida bipuncta (Hübner, 1824)
Maurica breveti (Oberthür, 1882)
Coscinia romeii Sagarra, 1924

Subfamily Herminiinae

Pechipogo simplicicornis (Zerny, 1935)
Herminia flavicrinalis Andreas, 1910

Subfamily Boletobiinae

Parascotia lorai Agenjo, 1967

Subfamily Eublemminae

Odice suava (Hübner, 1813) *
Eublemma albida (Duponchel, 1843)
Eublemma himmighoffeni (Millière, 1867)
Eublemma candicans (Rambur, 1858)

Subfamily Erebinae

Catocala diversa (Séller, 1828)
Catocala oberthueri Austaut, 1879
Cerocala scapulosa (Hübner, 1808)
Dysgonia torrida (Guenée, 1852)
