# Supplementary material for: Close congruence between Barcode Index Numbers (bins) and species boundaries in the Erebidae (Lepidoptera: Noctuoidea) of the Iberian Peninsula
Source: Biodivers Data J. 2017 Aug 8;(5):e19840. doi: 10.3897/BDJ.5.e19840 (PMC5558050; doi:10.3897/BDJ.5.e19840)
Supplement: Supplementary material 4 — BOLD TaxonID Tree [file bdj-05-e19840-s004.pdf]

# BOLD TaxonID Tree

| | | |
|---|---|---|
| Title | : | Iberian Erebidae Lepidoptera [DS-IBEREBID] |
| Date | : | 22-December-2016 |
| Data Type | : | Nucleotide |
| Distance Model | : | Kimura 2 Parameter |
| Marker | : | COI-5P |
| Codon Positions | : | 1st, 2nd, 3rd |
| Labels | : | Region, SampleID, BIN uri |
| Filters | : | Length > 500 |
| Colorization | : | [blue]=Stop Codons [red]=Contamination or misidentification |
| | | |
| Sequence Count | : | 357 |
| Species count | : | 161 |
| Genus count | : | 82 |
| Family count | : | 1 |
| Unidentified | : | 0 |
| | | |
| BIN Count | : | 164 |

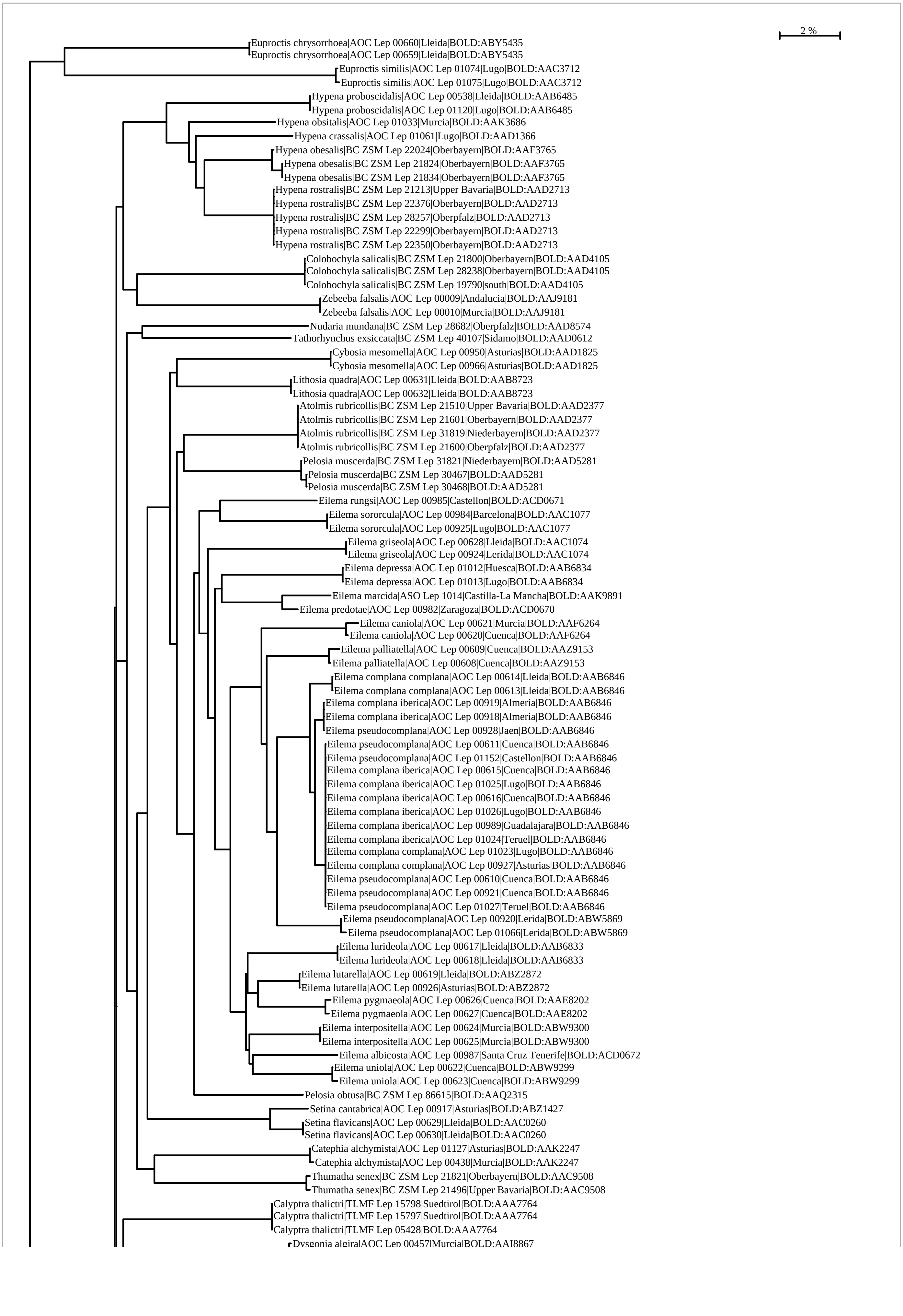
2 %
Euproctis chrysorrhoea|AOC Lep 00660|Lleida|BOLD:ABY5435
Euproctis chrysorrhoea|AOC Lep 00659|Lleida|BOLD:ABY5435
Euproctis similis|AOC Lep 01074|Lugo|BOLD:AAC3712
Euproctis similis|AOC Lep 01075|Lugo|BOLD:AAC3712
Hypena proboscidalis|AOC Lep 00538|Lleida|BOLD:AAB6485
Hypena proboscidalis|AOC Lep 01120|Lugo|BOLD:AAB6485
Hypena obsitalis|AOC Lep 01033|Murcia|BOLD:AAK3686
Hypena crassalis|AOC Lep 01061|Lugo|BOLD:AAD1366
Hypena obesalis|BC ZSM Lep 22024|Oberbayern|BOLD:AAF3765
Hypena obesalis|BC ZSM Lep 21824|Oberbayern|BOLD:AAF3765
Hypena obesalis|BC ZSM Lep 21834|Oberbayern|BOLD:AAF3765
Hypena rostralis|BC ZSM Lep 21213|Upper Bavaria|BOLD:AAD2713
Hypena rostralis|BC ZSM Lep 22376|Oberbayern|BOLD:AAD2713
Hypena rostralis|BC ZSM Lep 28257|Oberpfalz|BOLD:AAD2713
Hypena rostralis|BC ZSM Lep 22299|Oberbayern|BOLD:AAD2713
Hypena rostralis|BC ZSM Lep 22350|Oberbayern|BOLD:AAD2713
Colobochyla salicalis|BC ZSM Lep 21800|Oberbayern|BOLD:AAD4105
Colobochyla salicalis|BC ZSM Lep 28238|Oberbayern|BOLD:AAD4105
Colobochyla salicalis|BC ZSM Lep 19790|south|BOLD:AAD4105
Zebeeba falsalis|AOC Lep 00009|Andalucia|BOLD:AAJ9181
Zebeeba falsalis|AOC Lep 00010|Murcia|BOLD:AAJ9181
Nudaria mundana|BC ZSM Lep 28682|Oberpfalz|BOLD:AAD8574
Tathorhynchus exsiccata|BC ZSM Lep 40107|Sidamo|BOLD:AAD0612
Cybosia mesomella|AOC Lep 00950|Asturias|BOLD:AAD1825
Cybosia mesomella|AOC Lep 00966|Asturias|BOLD:AAD1825
Lithosia quadra|AOC Lep 00631|Lleida|BOLD:AAB8723
Lithosia quadra|AOC Lep 00632|Lleida|BOLD:AAB8723
Atolmis rubricollis|BC ZSM Lep 21510|Upper Bavaria|BOLD:AAD2377
Atolmis rubricollis|BC ZSM Lep 21601|Oberbayern|BOLD:AAD2377
Atolmis rubricollis|BC ZSM Lep 31819|Niederbayern|BOLD:AAD2377
Atolmis rubricollis|BC ZSM Lep 21600|Oberpfalz|BOLD:AAD2377
Pelosia muscerda|BC ZSM Lep 31821|Niederbayern|BOLD:AAD5281
Pelosia muscerda|BC ZSM Lep 30467|BOLD:AAD5281
Pelosia muscerda|BC ZSM Lep 30468|BOLD:AAD5281
Eilema rungsi|AOC Lep 00985|Castellon|BOLD:ACD0671
Eilema sororcula|AOC Lep 00984|Barcelona|BOLD:AAC1077
Eilema sororcula|AOC Lep 00925|Lugo|BOLD:AAC1077
Eilema griseola|AOC Lep 00628|Lleida|BOLD:AAC1074
Eilema griseola|AOC Lep 00924|Lerida|BOLD:AAC1074
Eilema depressa|AOC Lep 01012|Huesca|BOLD:AAB6834
Eilema depressa|AOC Lep 01013|Lugo|BOLD:AAB6834
Eilema marcida|ASO Lep 1014|Castilla-La Mancha|BOLD:AAK9891
Eilema predotae|AOC Lep 00982|Zaragoza|BOLD:ACD0670
Eilema caniola|AOC Lep 00621|Murcia|BOLD:AAF6264
Eilema caniola|AOC Lep 00620|Cuenca|BOLD:AAF6264
Eilema palliatella|AOC Lep 00609|Cuenca|BOLD:AAZ9153
Eilema palliatella|AOC Lep 00608|Cuenca|BOLD:AAZ9153
Eilema complana complana|AOC Lep 00614|Lleida|BOLD:AAB6846
Eilema complana complana|AOC Lep 00613|Lleida|BOLD:AAB6846
Eilema complana iberica|AOC Lep 00919|Almeria|BOLD:AAB6846
Eilema complana iberica|AOC Lep 00918|Almeria|BOLD:AAB6846
Eilema pseudocomplana|AOC Lep 00928|Jaen|BOLD:AAB6846
Eilema pseudocomplana|AOC Lep 00611|Cuenca|BOLD:AAB6846
Eilema pseudocomplana|AOC Lep 01152|Castellon|BOLD:AAB6846
Eilema complana iberica|AOC Lep 00615|Cuenca|BOLD:AAB6846
Eilema complana iberica|AOC Lep 01025|Lugo|BOLD:AAB6846
Eilema complana iberica|AOC Lep 00616|Cuenca|BOLD:AAB6846
Eilema complana iberica|AOC Lep 01026|Lugo|BOLD:AAB6846
Eilema complana iberica|AOC Lep 00989|Guadalajara|BOLD:AAB6846
Eilema complana iberica|AOC Lep 01024|Teruel|BOLD:AAB6846
Eilema complana complana|AOC Lep 01023|Lugo|BOLD:AAB6846
Eilema complana complana|AOC Lep 00927|Asturias|BOLD:AAB6846
Eilema pseudocomplana|AOC Lep 00610|Cuenca|BOLD:AAB6846
Eilema pseudocomplana|AOC Lep 00921|Cuenca|BOLD:AAB6846
Eilema pseudocomplana|AOC Lep 01027|Teruel|BOLD:AAB6846
Eilema pseudocomplana|AOC Lep 00920|Lerida|BOLD:ABW5869
Eilema pseudocomplana|AOC Lep 01066|Lerida|BOLD:ABW5869
Eilema lurideola|AOC Lep 00617|Lleida|BOLD:AAB6833
Eilema lurideola|AOC Lep 00618|Lleida|BOLD:AAB6833
Eilema lutarella|AOC Lep 00619|Lleida|BOLD:ABZ2872
Eilema lutarella|AOC Lep 00926|Asturias|BOLD:ABZ2872
Eilema pygmaeola|AOC Lep 00626|Cuenca|BOLD:AAE8202
Eilema pygmaeola|AOC Lep 00627|Cuenca|BOLD:AAE8202
Eilema interpositella|AOC Lep 00624|Murcia|BOLD:ABW9300
Eilema interpositella|AOC Lep 00625|Murcia|BOLD:ABW9300
Eilema albicosta|AOC Lep 00987|Santa Cruz Tenerife|BOLD:ACD0672
Eilema uniola|AOC Lep 00622|Cuenca|BOLD:ABW9299
Eilema uniola|AOC Lep 00623|Cuenca|BOLD:ABW9299
Pelosia obtusa|BC ZSM Lep 86615|BOLD:AAQ2315
Setina cantabrica|AOC Lep 00917|Asturias|BOLD:ABZ1427
Setina flavicans|AOC Lep 00629|Lleida|BOLD:AAC0260
Setina flavicans|AOC Lep 00630|Lleida|BOLD:AAC0260
Catephia alchymista|AOC Lep 01127|Asturias|BOLD:AAK2247
Catephia alchymista|AOC Lep 00438|Murcia|BOLD:AAK2247
Thumatha senex|BC ZSM Lep 21821|Oberbayern|BOLD:AAC9508
Thumatha senex|BC ZSM Lep 21496|Upper Bavaria|BOLD:AAC9508
Calyptra thalictri|TLMF Lep 15798|Suedtirol|BOLD:AAA7764
Calyptra thalictri|TLMF Lep 15797|Suedtirol|BOLD:AAA7764
Calyptra thalictri|TLMF Lep 05428|BOLD:AAA7764
Dysgonia algira|AOC Lep 00457|Murcia|BOLD:AAI8867

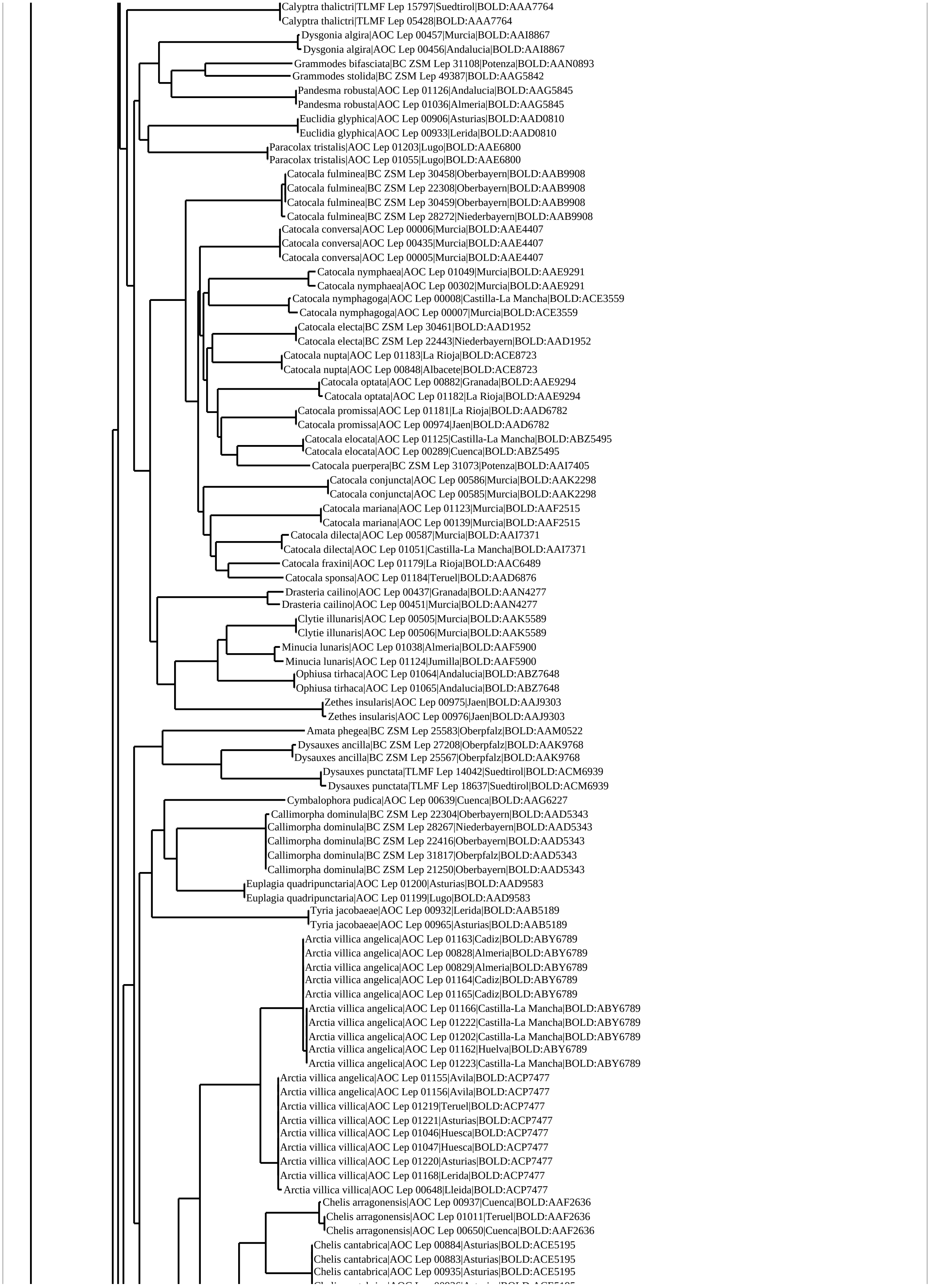
Calyptra thalictri|TLMF Lep 15797|Suedtirol|BOLD:AAA7764
Calyptra thalictri|TLMF Lep 05428|BOLD:AAA7764
Dysgonia algira|AOC Lep 00457|Murcia|BOLD:AAI8867
Dysgonia algira|AOC Lep 00456|Andalucia|BOLD:AAI8867
Grammodes bifasciata|BC ZSM Lep 31108|Potenza|BOLD:AAN0893
Grammodes stolida|BC ZSM Lep 49387|BOLD:AAG5842
Pandesma robusta|AOC Lep 01126|Andalucia|BOLD:AAG5845
Pandesma robusta|AOC Lep 01036|Almeria|BOLD:AAG5845
Euclidia glyphica|AOC Lep 00906|Asturias|BOLD:AAD0810
Euclidia glyphica|AOC Lep 00933|Lerida|BOLD:AAD0810
Paracolax tristalis|AOC Lep 01203|Lugo|BOLD:AAE6800
Paracolax tristalis|AOC Lep 01055|Lugo|BOLD:AAE6800
Catocala fulminea|BC ZSM Lep 30458|Oberbayern|BOLD:AAB9908
Catocala fulminea|BC ZSM Lep 22308|Oberbayern|BOLD:AAB9908
Catocala fulminea|BC ZSM Lep 30459|Oberbayern|BOLD:AAB9908
Catocala fulminea|BC ZSM Lep 28272|Niederbayern|BOLD:AAB9908
Catocala conversa|AOC Lep 00006|Murcia|BOLD:AAE4407
Catocala conversa|AOC Lep 00435|Murcia|BOLD:AAE4407
Catocala conversa|AOC Lep 00005|Murcia|BOLD:AAE4407
Catocala nymphaea|AOC Lep 01049|Murcia|BOLD:AAE9291
Catocala nymphaea|AOC Lep 00302|Murcia|BOLD:AAE9291
Catocala nymphagoga|AOC Lep 00008|Castilla-La Mancha|BOLD:ACE3559
Catocala nymphagoga|AOC Lep 00007|Murcia|BOLD:ACE3559
Catocala electa|BC ZSM Lep 30461|BOLD:AAD1952
Catocala electa|BC ZSM Lep 22443|Niederbayern|BOLD:AAD1952
Catocala nupta|AOC Lep 01183|La Rioja|BOLD:ACE8723
Catocala nupta|AOC Lep 00848|Albacete|BOLD:ACE8723
Catocala optata|AOC Lep 00882|Granada|BOLD:AAE9294
Catocala optata|AOC Lep 01182|La Rioja|BOLD:AAE9294
Catocala promissa|AOC Lep 01181|La Rioja|BOLD:AAD6782
Catocala promissa|AOC Lep 00974|Jaen|BOLD:AAD6782
Catocala elocata|AOC Lep 01125|Castilla-La Mancha|BOLD:ABZ5495
Catocala elocata|AOC Lep 00289|Cuenca|BOLD:ABZ5495
Catocala puerpera|BC ZSM Lep 31073|Potenza|BOLD:AAI7405
Catocala conjuncta|AOC Lep 00586|Murcia|BOLD:AAK2298
Catocala conjuncta|AOC Lep 00585|Murcia|BOLD:AAK2298
Catocala mariana|AOC Lep 01123|Murcia|BOLD:AAF2515
Catocala mariana|AOC Lep 00139|Murcia|BOLD:AAF2515
Catocala dilecta|AOC Lep 00587|Murcia|BOLD:AAI7371
Catocala dilecta|AOC Lep 01051|Castilla-La Mancha|BOLD:AAI7371
Catocala fraxini|AOC Lep 01179|La Rioja|BOLD:AAC6489
Catocala sponsa|AOC Lep 01184|Teruel|BOLD:AAD6876
Drasteria cailino|AOC Lep 00437|Granada|BOLD:AAN4277
Drasteria cailino|AOC Lep 00451|Murcia|BOLD:AAN4277
Clytie illunaris|AOC Lep 00505|Murcia|BOLD:AAK5589
Clytie illunaris|AOC Lep 00506|Murcia|BOLD:AAK5589
Minucia lunaris|AOC Lep 01038|Almeria|BOLD:AAF5900
Minucia lunaris|AOC Lep 01124|Jumilla|BOLD:AAF5900
Ophiusa tirhaca|AOC Lep 01064|Andalucia|BOLD:ABZ7648
Ophiusa tirhaca|AOC Lep 01065|Andalucia|BOLD:ABZ7648
Zethes insularis|AOC Lep 00975|Jaen|BOLD:AAJ9303
Zethes insularis|AOC Lep 00976|Jaen|BOLD:AAJ9303
Amata phegea|BC ZSM Lep 25583|Oberpfalz|BOLD:AAM0522
Dysauxes ancilla|BC ZSM Lep 27208|Oberpfalz|BOLD:AAK9768
Dysauxes ancilla|BC ZSM Lep 25567|Oberpfalz|BOLD:AAK9768
Dysauxes punctata|TLMF Lep 14042|Suedtirol|BOLD:ACM6939
Dysauxes punctata|TLMF Lep 18637|Suedtirol|BOLD:ACM6939
Cymbalophora pudica|AOC Lep 00639|Cuenca|BOLD:AAG6227
Callimorpha dominula|BC ZSM Lep 22304|Oberbayern|BOLD:AAD5343
Callimorpha dominula|BC ZSM Lep 28267|Niederbayern|BOLD:AAD5343
Callimorpha dominula|BC ZSM Lep 22416|Oberbayern|BOLD:AAD5343
Callimorpha dominula|BC ZSM Lep 31817|Oberpfalz|BOLD:AAD5343
Callimorpha dominula|BC ZSM Lep 21250|Oberbayern|BOLD:AAD5343
Euplagia quadripunctaria|AOC Lep 01200|Asturias|BOLD:AAD9583
Euplagia quadripunctaria|AOC Lep 01199|Lugo|BOLD:AAD9583
Tyria jacobaeae|AOC Lep 00932|Lerida|BOLD:AAB5189
Tyria jacobaeae|AOC Lep 00965|Asturias|BOLD:AAB5189
Arctia villica angelica|AOC Lep 01163|Cadiz|BOLD:ABY6789
Arctia villica angelica|AOC Lep 00828|Almeria|BOLD:ABY6789
Arctia villica angelica|AOC Lep 00829|Almeria|BOLD:ABY6789
Arctia villica angelica|AOC Lep 01164|Cadiz|BOLD:ABY6789
Arctia villica angelica|AOC Lep 01165|Cadiz|BOLD:ABY6789
Arctia villica angelica|AOC Lep 01166|Castilla-La Mancha|BOLD:ABY6789
Arctia villica angelica|AOC Lep 01222|Castilla-La Mancha|BOLD:ABY6789
Arctia villica angelica|AOC Lep 01202|Castilla-La Mancha|BOLD:ABY6789
Arctia villica angelica|AOC Lep 01162|Huelva|BOLD:ABY6789
Arctia villica angelica|AOC Lep 01223|Castilla-La Mancha|BOLD:ABY6789
Arctia villica angelica|AOC Lep 01155|Avila|BOLD:ACP7477
Arctia villica angelica|AOC Lep 01156|Avila|BOLD:ACP7477
Arctia villica villica|AOC Lep 01219|Teruel|BOLD:ACP7477
Arctia villica villica|AOC Lep 01221|Asturias|BOLD:ACP7477
Arctia villica villica|AOC Lep 01046|Huesca|BOLD:ACP7477
Arctia villica villica|AOC Lep 01047|Huesca|BOLD:ACP7477
Arctia villica villica|AOC Lep 01220|Asturias|BOLD:ACP7477
Arctia villica villica|AOC Lep 01168|Lerida|BOLD:ACP7477
Arctia villica villica|AOC Lep 00648|Lleida|BOLD:ACP7477
Chelis arragonensis|AOC Lep 00937|Cuenca|BOLD:AAF2636
Chelis arragonensis|AOC Lep 01011|Teruel|BOLD:AAF2636
Chelis arragonensis|AOC Lep 00650|Cuenca|BOLD:AAF2636
Chelis cantabrica|AOC Lep 00884|Asturias|BOLD:ACE5195
Chelis cantabrica|AOC Lep 00883|Asturias|BOLD:ACE5195
Chelis cantabrica|AOC Lep 00935|Asturias|BOLD:ACE5195

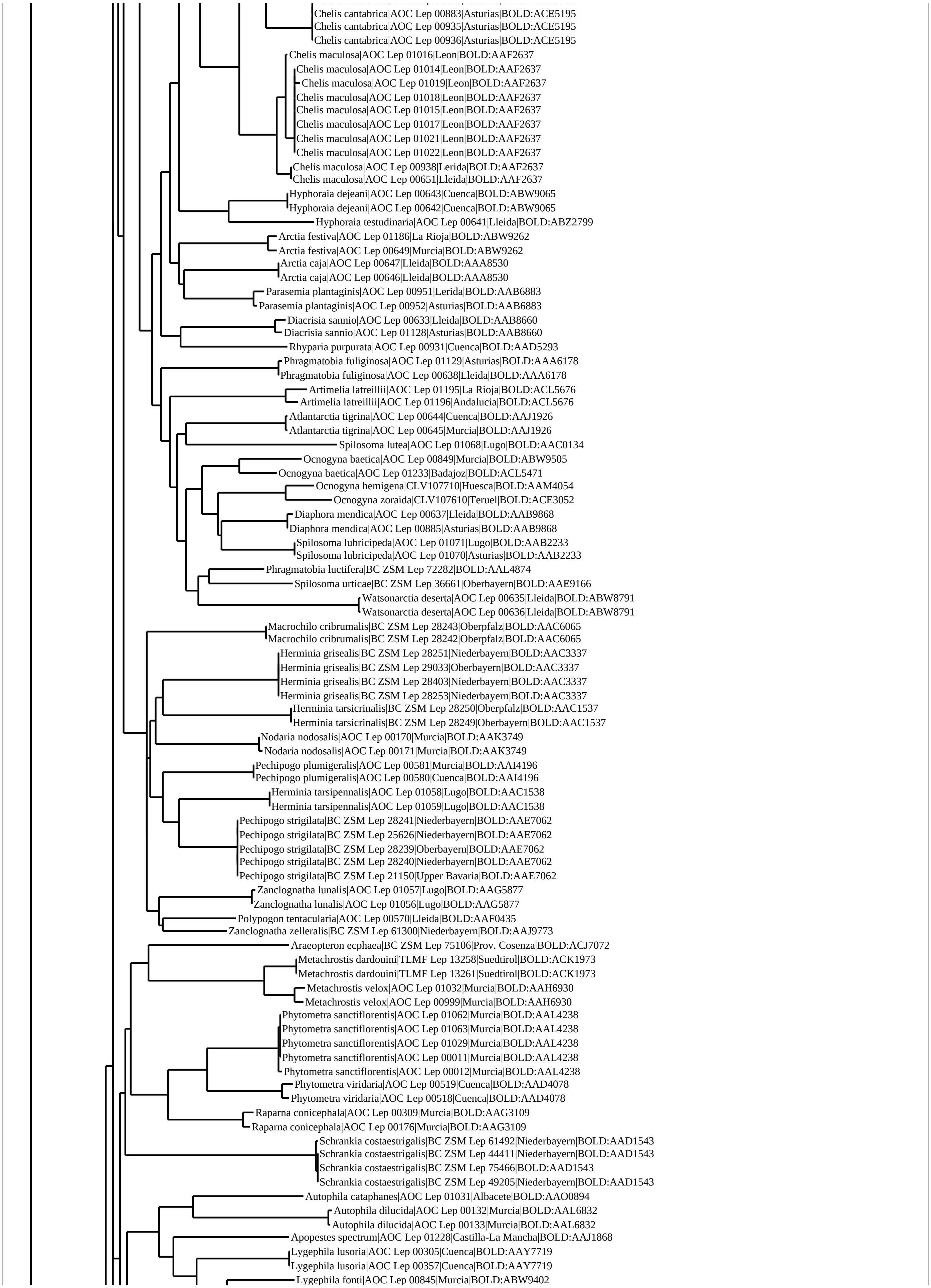
Chelis cantabrica|AOC Lep 00883|Asturias|BOLD:ACE5195
Chelis cantabrica|AOC Lep 00935|Asturias|BOLD:ACE5195
Chelis cantabrica|AOC Lep 00936|Asturias|BOLD:ACE5195
Chelis maculosa|AOC Lep 01016|Leon|BOLD:AAF2637
Chelis maculosa|AOC Lep 01014|Leon|BOLD:AAF2637
Chelis maculosa|AOC Lep 01019|Leon|BOLD:AAF2637
Chelis maculosa|AOC Lep 01018|Leon|BOLD:AAF2637
Chelis maculosa|AOC Lep 01015|Leon|BOLD:AAF2637
Chelis maculosa|AOC Lep 01017|Leon|BOLD:AAF2637
Chelis maculosa|AOC Lep 01021|Leon|BOLD:AAF2637
Chelis maculosa|AOC Lep 01022|Leon|BOLD:AAF2637
Chelis maculosa|AOC Lep 00938|Lerida|BOLD:AAF2637
Chelis maculosa|AOC Lep 00651|Lleida|BOLD:AAF2637
Hyphoraia dejeani|AOC Lep 00643|Cuenca|BOLD:ABW9065
Hyphoraia dejeani|AOC Lep 00642|Cuenca|BOLD:ABW9065
Hyphoraia testudinaria|AOC Lep 00641|Lleida|BOLD:ABZ2799
Arctia festiva|AOC Lep 01186|La Rioja|BOLD:ABW9262
Arctia festiva|AOC Lep 00649|Murcia|BOLD:ABW9262
Arctia caja|AOC Lep 00647|Lleida|BOLD:AAA8530
Arctia caja|AOC Lep 00646|Lleida|BOLD:AAA8530
Parasemia plantaginis|AOC Lep 00951|Lerida|BOLD:AAB6883
Parasemia plantaginis|AOC Lep 00952|Asturias|BOLD:AAB6883
Diacrisia sannio|AOC Lep 00633|Lleida|BOLD:AAB8660
Diacrisia sannio|AOC Lep 01128|Asturias|BOLD:AAB8660
Rhyparia purpurata|AOC Lep 00931|Cuenca|BOLD:AAD5293
Phragmatobia fuliginosa|AOC Lep 01129|Asturias|BOLD:AAA6178
Phragmatobia fuliginosa|AOC Lep 00638|Lleida|BOLD:AAA6178
Artimelia latreillii|AOC Lep 01195|La Rioja|BOLD:ACL5676
Artimelia latreillii|AOC Lep 01196|Andalucia|BOLD:ACL5676
Atlantarctia tigrina|AOC Lep 00644|Cuenca|BOLD:AAJ1926
Atlantarctia tigrina|AOC Lep 00645|Murcia|BOLD:AAJ1926
Spilosoma lutea|AOC Lep 01068|Lugo|BOLD:AAC0134
Ocnogyna baetica|AOC Lep 00849|Murcia|BOLD:ABW9505
Ocnogyna baetica|AOC Lep 01233|Badajoz|BOLD:ACL5471
Ocnogyna hemigena|CLV107710|Huesca|BOLD:AAM4054
Ocnogyna zoraida|CLV107610|Teruel|BOLD:ACE3052
Diaphora mendica|AOC Lep 00637|Lleida|BOLD:AAB9868
Diaphora mendica|AOC Lep 00885|Asturias|BOLD:AAB9868
Spilosoma lubricipeda|AOC Lep 01071|Lugo|BOLD:AAB2233
Spilosoma lubricipeda|AOC Lep 01070|Asturias|BOLD:AAB2233
Phragmatobia luctifera|BC ZSM Lep 72282|BOLD:AAL4874
Spilosoma urticae|BC ZSM Lep 36661|Oberbayern|BOLD:AAE9166
Watsonarctia deserta|AOC Lep 00635|Lleida|BOLD:ABW8791
Watsonarctia deserta|AOC Lep 00636|Lleida|BOLD:ABW8791
Macrochilo cribrumalis|BC ZSM Lep 28243|Oberpfalz|BOLD:AAC6065
Macrochilo cribrumalis|BC ZSM Lep 28242|Oberpfalz|BOLD:AAC6065
Herminia grisealis|BC ZSM Lep 28251|Niederbayern|BOLD:AAC3337
Herminia grisealis|BC ZSM Lep 29033|Oberbayern|BOLD:AAC3337
Herminia grisealis|BC ZSM Lep 28403|Niederbayern|BOLD:AAC3337
Herminia grisealis|BC ZSM Lep 28253|Niederbayern|BOLD:AAC3337
Herminia tarsicrinalis|BC ZSM Lep 28250|Oberpfalz|BOLD:AAC1537
Herminia tarsicrinalis|BC ZSM Lep 28249|Oberbayern|BOLD:AAC1537
Nodaria nodosalis|AOC Lep 00170|Murcia|BOLD:AAK3749
Nodaria nodosalis|AOC Lep 00171|Murcia|BOLD:AAK3749
Pechipogo plumigeralis|AOC Lep 00581|Murcia|BOLD:AAI4196
Pechipogo plumigeralis|AOC Lep 00580|Cuenca|BOLD:AAI4196
Herminia tarsipennalis|AOC Lep 01058|Lugo|BOLD:AAC1538
Herminia tarsipennalis|AOC Lep 01059|Lugo|BOLD:AAC1538
Pechipogo strigilata|BC ZSM Lep 28241|Niederbayern|BOLD:AAE7062
Pechipogo strigilata|BC ZSM Lep 25626|Niederbayern|BOLD:AAE7062
Pechipogo strigilata|BC ZSM Lep 28239|Oberbayern|BOLD:AAE7062
Pechipogo strigilata|BC ZSM Lep 28240|Niederbayern|BOLD:AAE7062
Pechipogo strigilata|BC ZSM Lep 21150|Upper Bavaria|BOLD:AAE7062
Zanclognatha lunalis|AOC Lep 01057|Lugo|BOLD:AAG5877
Zanclognatha lunalis|AOC Lep 01056|Lugo|BOLD:AAG5877
Polypogon tentacularia|AOC Lep 00570|Lleida|BOLD:AAF0435
Zanclognatha zelleralis|BC ZSM Lep 61300|Niederbayern|BOLD:AAJ9773
Araeopteron ecphaea|BC ZSM Lep 75106|Prov. Cosenza|BOLD:ACJ7072
Metachrostis dardouini|TLMF Lep 13258|Suedtirol|BOLD:ACK1973
Metachrostis dardouini|TLMF Lep 13261|Suedtirol|BOLD:ACK1973
Metachrostis velox|AOC Lep 01032|Murcia|BOLD:AAH6930
Metachrostis velox|AOC Lep 00999|Murcia|BOLD:AAH6930
Phytometra sanctiflorentis|AOC Lep 01062|Murcia|BOLD:AAL4238
Phytometra sanctiflorentis|AOC Lep 01063|Murcia|BOLD:AAL4238
Phytometra sanctiflorentis|AOC Lep 01029|Murcia|BOLD:AAL4238
Phytometra sanctiflorentis|AOC Lep 00011|Murcia|BOLD:AAL4238
Phytometra sanctiflorentis|AOC Lep 00012|Murcia|BOLD:AAL4238
Phytometra viridaria|AOC Lep 00519|Cuenca|BOLD:AAD4078
Phytometra viridaria|AOC Lep 00518|Cuenca|BOLD:AAD4078
Raparna conicephala|AOC Lep 00309|Murcia|BOLD:AAG3109
Raparna conicephala|AOC Lep 00176|Murcia|BOLD:AAG3109
Schrankia costaestrigalis|BC ZSM Lep 61492|Niederbayern|BOLD:AAD1543
Schrankia costaestrigalis|BC ZSM Lep 44411|Niederbayern|BOLD:AAD1543
Schrankia costaestrigalis|BC ZSM Lep 75466|BOLD:AAD1543
Schrankia costaestrigalis|BC ZSM Lep 49205|Niederbayern|BOLD:AAD1543
Autophila cataphanes|AOC Lep 01031|Albacete|BOLD:AAO0894
Autophila dilucida|AOC Lep 00132|Murcia|BOLD:AAL6832
Autophila dilucida|AOC Lep 00133|Murcia|BOLD:AAL6832
Apopestes spectrum|AOC Lep 01228|Castilla-La Mancha|BOLD:AAJ1868
Lygephila lusoria|AOC Lep 00305|Cuenca|BOLD:AAY7719
Lygephila lusoria|AOC Lep 00357|Cuenca|BOLD:AAY7719
Lygephila fonti|AOC Lep 00845|Murcia|BOLD:ABW9402

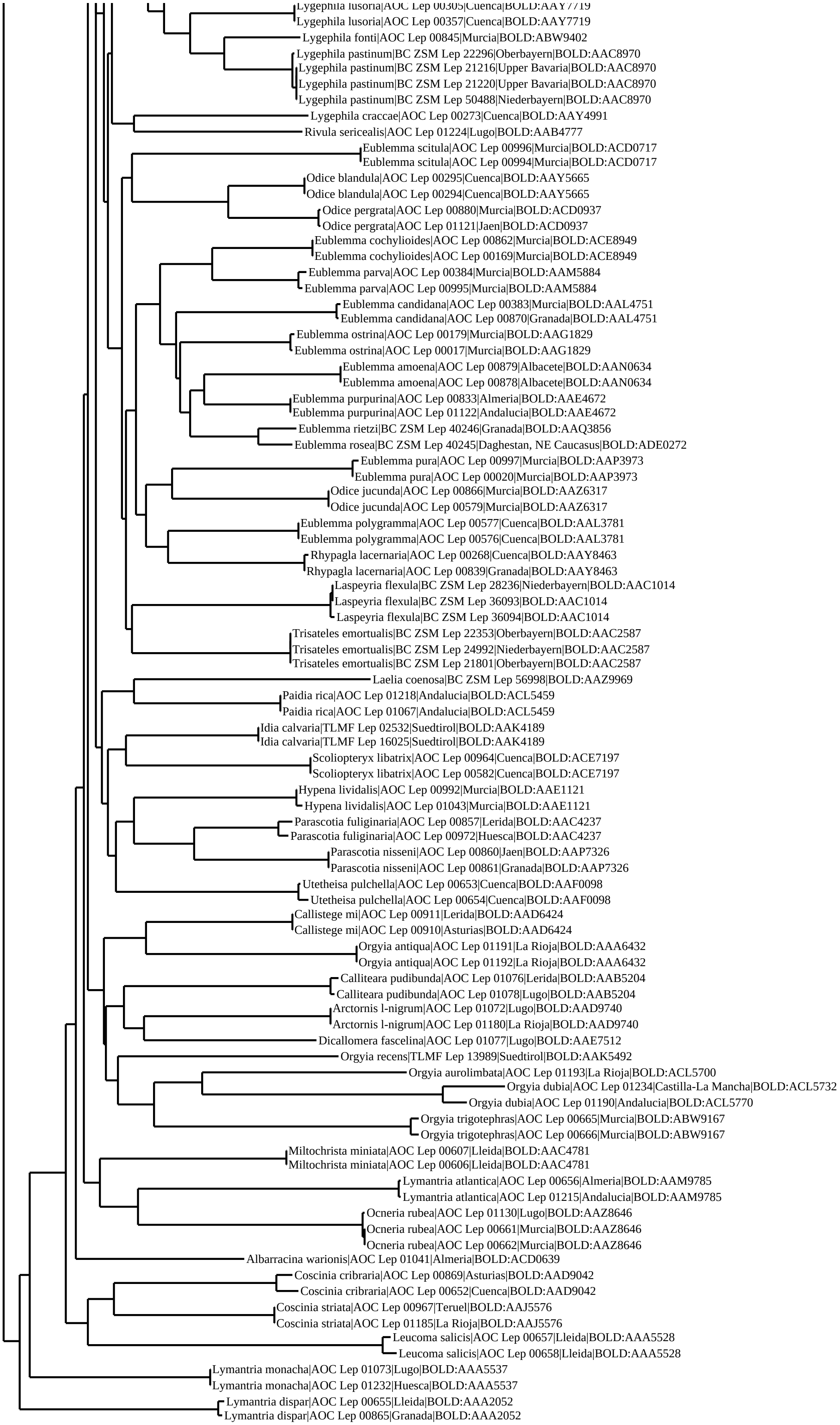
Lygephila lusoria|AOC Lep 00305|Cuenca|BOLD:AAY7719
Lygephila lusoria|AOC Lep 00357|Cuenca|BOLD:AAY7719
Lygephila fonti|AOC Lep 00845|Murcia|BOLD:ABW9402
Lygephila pastinum|BC ZSM Lep 22296|Oberbayern|BOLD:AAC8970
Lygephila pastinum|BC ZSM Lep 21216|Upper Bavaria|BOLD:AAC8970
Lygephila pastinum|BC ZSM Lep 21220|Upper Bavaria|BOLD:AAC8970
Lygephila pastinum|BC ZSM Lep 50488|Niederbayern|BOLD:AAC8970
Lygephila craccae|AOC Lep 00273|Cuenca|BOLD:AAY4991
Rivula sericealis|AOC Lep 01224|Lugo|BOLD:AAB4777
Eublemma scitula|AOC Lep 00996|Murcia|BOLD:ACD0717
Eublemma scitula|AOC Lep 00994|Murcia|BOLD:ACD0717
Odice blandula|AOC Lep 00295|Cuenca|BOLD:AAY5665
Odice blandula|AOC Lep 00294|Cuenca|BOLD:AAY5665
Odice pergrata|AOC Lep 00880|Murcia|BOLD:ACD0937
Odice pergrata|AOC Lep 01121|Jaen|BOLD:ACD0937
Eublemma cochylioides|AOC Lep 00862|Murcia|BOLD:ACE8949
Eublemma cochylioides|AOC Lep 00169|Murcia|BOLD:ACE8949
Eublemma parva|AOC Lep 00384|Murcia|BOLD:AAM5884
Eublemma parva|AOC Lep 00995|Murcia|BOLD:AAM5884
Eublemma candidana|AOC Lep 00383|Murcia|BOLD:AAL4751
Eublemma candidana|AOC Lep 00870|Granada|BOLD:AAL4751
Eublemma ostrina|AOC Lep 00179|Murcia|BOLD:AAG1829
Eublemma ostrina|AOC Lep 00017|Murcia|BOLD:AAG1829
Eublemma amoena|AOC Lep 00879|Albacete|BOLD:AAN0634
Eublemma amoena|AOC Lep 00878|Albacete|BOLD:AAN0634
Eublemma purpurina|AOC Lep 00833|Almeria|BOLD:AAE4672
Eublemma purpurina|AOC Lep 01122|Andalucia|BOLD:AAE4672
Eublemma rietzi|BC ZSM Lep 40246|Granada|BOLD:AAQ3856
Eublemma rosea|BC ZSM Lep 40245|Daghestan, NE Caucasus|BOLD:ADE0272
Eublemma pura|AOC Lep 00997|Murcia|BOLD:AAP3973
Eublemma pura|AOC Lep 00020|Murcia|BOLD:AAP3973
Odice jucunda|AOC Lep 00866|Murcia|BOLD:AAZ6317
Odice jucunda|AOC Lep 00579|Murcia|BOLD:AAZ6317
Eublemma polygramma|AOC Lep 00577|Cuenca|BOLD:AAL3781
Eublemma polygramma|AOC Lep 00576|Cuenca|BOLD:AAL3781
Rhypagla lacernaria|AOC Lep 00268|Cuenca|BOLD:AAY8463
Rhypagla lacernaria|AOC Lep 00839|Granada|BOLD:AAY8463
Laspeyria flexula|BC ZSM Lep 28236|Niederbayern|BOLD:AAC1014
Laspeyria flexula|BC ZSM Lep 36093|BOLD:AAC1014
Laspeyria flexula|BC ZSM Lep 36094|BOLD:AAC1014
Trisateles emortualis|BC ZSM Lep 22353|Oberbayern|BOLD:AAC2587
Trisateles emortualis|BC ZSM Lep 24992|Niederbayern|BOLD:AAC2587
Trisateles emortualis|BC ZSM Lep 21801|Oberbayern|BOLD:AAC2587
Laelia coenosa|BC ZSM Lep 56998|BOLD:AAZ9969
Paidia rica|AOC Lep 01218|Andalucia|BOLD:ACL5459
Paidia rica|AOC Lep 01067|Andalucia|BOLD:ACL5459
Idia calvaria|TLMF Lep 02532|Suedtirol|BOLD:AAK4189
Idia calvaria|TLMF Lep 16025|Suedtirol|BOLD:AAK4189
Scoliopteryx libatrix|AOC Lep 00964|Cuenca|BOLD:ACE7197
Scoliopteryx libatrix|AOC Lep 00582|Cuenca|BOLD:ACE7197
Hypena lividalis|AOC Lep 00992|Murcia|BOLD:AAE1121
Hypena lividalis|AOC Lep 01043|Murcia|BOLD:AAE1121
Parascotia fuliginaria|AOC Lep 00857|Lerida|BOLD:AAC4237
Parascotia fuliginaria|AOC Lep 00972|Huesca|BOLD:AAC4237
Parascotia nisseni|AOC Lep 00860|Jaen|BOLD:AAP7326
Parascotia nisseni|AOC Lep 00861|Granada|BOLD:AAP7326
Utetheisa pulchella|AOC Lep 00653|Cuenca|BOLD:AAF0098
Utetheisa pulchella|AOC Lep 00654|Cuenca|BOLD:AAF0098
Callistege mi|AOC Lep 00911|Lerida|BOLD:AAD6424
Callistege mi|AOC Lep 00910|Asturias|BOLD:AAD6424
Orgyia antiqua|AOC Lep 01191|La Rioja|BOLD:AAA6432
Orgyia antiqua|AOC Lep 01192|La Rioja|BOLD:AAA6432
Calliteara pudibunda|AOC Lep 01076|Lerida|BOLD:AAB5204
Calliteara pudibunda|AOC Lep 01078|Lugo|BOLD:AAB5204
Arctornis l-nigrum|AOC Lep 01072|Lugo|BOLD:AAD9740
Arctornis l-nigrum|AOC Lep 01180|La Rioja|BOLD:AAD9740
Dicallomera fascelina|AOC Lep 01077|Lugo|BOLD:AAE7512
Orgyia recens|TLMF Lep 13989|Suedtirol|BOLD:AAK5492
Orgyia aurolimbata|AOC Lep 01193|La Rioja|BOLD:ACL5700
Orgyia dubia|AOC Lep 01234|Castilla-La Mancha|BOLD:ACL5732
Orgyia dubia|AOC Lep 01190|Andalucia|BOLD:ACL5770
Orgyia trigotephras|AOC Lep 00665|Murcia|BOLD:ABW9167
Orgyia trigotephras|AOC Lep 00666|Murcia|BOLD:ABW9167
Miltochrista miniata|AOC Lep 00607|Lleida|BOLD:AAC4781
Miltochrista miniata|AOC Lep 00606|Lleida|BOLD:AAC4781
Lymantria atlantica|AOC Lep 00656|Almeria|BOLD:AAM9785
Lymantria atlantica|AOC Lep 01215|Andalucia|BOLD:AAM9785
Ocneria rubea|AOC Lep 01130|Lugo|BOLD:AAZ8646
Ocneria rubea|AOC Lep 00661|Murcia|BOLD:AAZ8646
Ocneria rubea|AOC Lep 00662|Murcia|BOLD:AAZ8646
Albarracina warionis|AOC Lep 01041|Almeria|BOLD:ACD0639
Coscinia cribraria|AOC Lep 00869|Asturias|BOLD:AAD9042
Coscinia cribraria|AOC Lep 00652|Cuenca|BOLD:AAD9042
Coscinia striata|AOC Lep 00967|Teruel|BOLD:AAJ5576
Coscinia striata|AOC Lep 01185|La Rioja|BOLD:AAJ5576
Leucoma salicis|AOC Lep 00657|Lleida|BOLD:AAA5528
Leucoma salicis|AOC Lep 00658|Lleida|BOLD:AAA5528
Lymantria monacha|AOC Lep 01073|Lugo|BOLD:AAA5537
Lymantria monacha|AOC Lep 01232|Huesca|BOLD:AAA5537
Lymantria dispar|AOC Lep 00655|Lleida|BOLD:AAA2052
Lymantria dispar|AOC Lep 00865|Granada|BOLD:AAA2052
